# Inhibitor of Growth 4 (ING4) is a positive regulator of rRNA synthesis

**DOI:** 10.1038/s41598-019-53767-1

**Published:** 2019-11-21

**Authors:** Duc-Anh Trinh, Ryutaro Shirakawa, Tomohiro Kimura, Natsumi Sakata, Kota Goto, Hisanori Horiuchi

**Affiliations:** 1 Department of Oral Cancer Therapeutics, Graduate School of Dentistry, Tohoku University, Sendai, Japan; 2 Department of Molecular and Cellular Biology, Institute of Development, Aging and Cancer, Tohoku University, Sendai, Japan; 3 Present Address: Research Center for Molecular Genetics, Institute for Promotion of Medical Science Research, Yamagata University Faculty of Medicine, Yamagata, Yamagata, Japan

**Keywords:** RNA, Stress signalling, Histone post-translational modifications, Nucleolus

## Abstract

Ribosome biogenesis is essential for maintaining basic cellular activities although its mechanism is not fully understood. Inhibitor of growth 4 (ING4) is a member of ING family while its cellular functions remain controversial. Here, we identified several nucleolar proteins as novel ING4 interacting proteins. ING4 localized in the nucleus with strong accumulation in the nucleolus through its plant homeodomain, which is known to interact with histone trimethylated H3K4, commonly present in the promoter of active genes. ING4 deficient cells exhibited slower proliferation and the alteration in nucleolar structure with reduced rRNA transcription, which was rescued by exogenous expression of GFP-ING4 to the similar levels of wild type cells. In the ING4 deficient cells, histone H3K9 acetylation and the key rRNA transcription factor UBF at the promoter of rDNA were reduced, both of which were also recovered by exogenous GFP-ING4 expression. Thus, ING4 could positively regulate rRNA transcription through modulation of histone modifications at the rDNA promoter.

## Introduction

Inhibitor of Growth (ING) protein family is composed of 5 members, which share a highly conserved homologous plant-homeodomain (PHD) at their C-terminus. PHD domain has emerged as a binding domain of trimethylated histone H3 lysine 4 (H3K4me3)^1^, commonly present in the promoter region of an active gene^2^. ING1, the prototype member of ING family, has first been demonstrated to inhibit cell proliferation^3^. Considering their structural similarity, all ING members had been proposed to be common in inhibition of cell growth and function as tumor suppressors^4,5^. Recently, however, conflicting data have been reported for ING3-5 in the regulation of cell proliferation^6–9^. As for the function of ING4 in cell proliferation, both positive and negative regulations have so far been reported. ING4 has been reported to bind and activate p53, resulting in induction of p21 tumor suppressor and inhibition of cell proliferation^10,11^. It has also been reported to inhibit cell proliferation by affecting NF-κB^12^. On the other hand, it has been reported to enhance tumor cell growth *in vitro* and *in vivo*^13^. Thus, the role of ING4 in the cell proliferation remains controversial.

In growing cells, a half of transcription is to generate ribosomal RNA (rRNA) and 70–80% ATP is consumed to produce ribosomes in a process called ribosome biogenesis^14,15^. The early steps of the process, which include rRNA transcription, processing and assembling with ribosomal proteins occur in the nucleolus. Later, complexes of rRNA and ribosomal proteins are exported into cytosol through nucleoplasm. rRNA transcription is exclusively catalyzed by RNA polymerase I (Pol I) to generate single 47S precursor ribosomal RNA (pre-rRNA)^15^. In turn, Pol I is under regulation of a wide range of biological molecules. Among them, upstream binding factor (UBF) is considered the most prominent transcription factor because its binding to the rDNA promoter is critical for recruitment of the pre-initiation complex and Pol I activity^16–18^.

Here, we identified several nucleolar proteins as novel ING4 binding proteins, and demonstrated that ING4 promoted cell proliferation and rRNA synthesis through modulating histone modifications at rDNA promoters.

## Results

### ING4 was associated with nucleolar proteins

**Figure 1:**
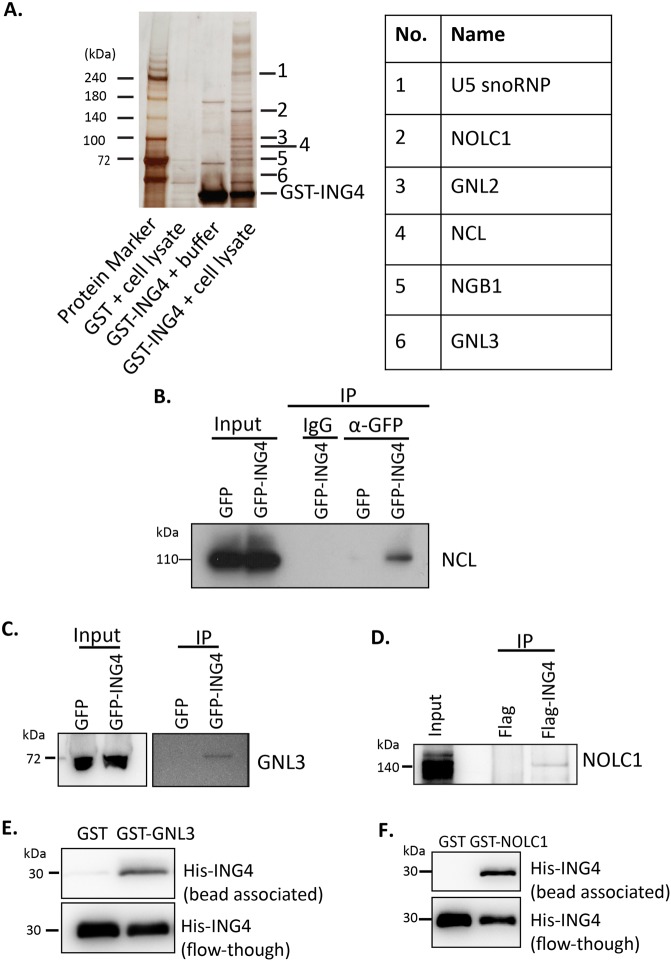
ING4 interacted with nucleolar proteins. (**A**) The affinity assay was performed with recombinant GST-ING4 and the HeLa S3 cell lysate as described in the Methods. Candidate proteins from the silver-stained SDS-PAGE were identified by nanoLC/MS/MS system. Identified nucleolar proteins are listed on the table. (**B**) *In vivo* interaction between ING4 and NCL. The pull-down analysis with control IgG or anti-GFP antibody was conducted with the lysate of HAP1 cells stably expressing either GFP or GFP-ING4. Aliquots of lysates from cells that stably expressed GFP or GFP-ING4 were used as inputs. Bead-associated proteins were analyzed by the western blot with anti-nucleolin antibody. (**C**) *In vivo* interaction between ING4 and GNL3. Similar to (**B**), the lysate from HAPI cells was incubated with nanobody beads against GFP. The subsequent western blot was analyzed with anti-GNL3 antibody. (**D**) *In vivo* interaction between ING4 and NOLC1. Here, the lysate from HEK293T cells that temporarily expressed Flag or Flag-ING4 was incubated with anti-Flag antibody and immobilized with protein G agarose. Bead-associated proteins were analyzed by the western blot with anti-NOLC1 antibody (**E**,**F**) *In vitro* interaction between either GST-GNL3 (**E**) or GST-NOLC1 (**F**) and His-ING4. GST or GST-GNL3 or GST-NOLC1 beads were incubated with His-ING4. Elution was conducted with 10 mM reduced glutathione solution. Eluate (upper lane) and flow-through (lower lane) were evaluated using western blot with anti-His antibody for His-ING4.

We first examined ING4 interacting proteins in HeLa S3 cell lysate by the affinity pull-down. Among the ING4-associated proteins identified, several nucleolar proteins such as nucleolar and coiled-body phosphoprotein 1 (NOLC1) that is known important in rRNA transcription^19^, nucleolar GTP-binding protein 2 (GNL2) that is important in the rRNA assembly and export^20^, G Protein nucleolar 3 (GNL3) that is important in rRNA processing^21^, nucleolar GTP-binding protein 1 (NGB1) and nucleolin (NCL), a critical regulator of rRNA transcription^22,23^, were included (Fig. 1A).

NCL was specifically co-immunoprecipitated with green fluorescent protein (GFP)-ING4, but not GFP, in stably expressing HAP1 cells (Fig. 1B). A similar result was obtained when we examined the interaction between GFP-ING4 and endogenous GNL3 (Fig. 1C). We also found the interaction between Flag-ING4 and endogenous NOLC1, or GNL2 in Flag-ING4 overexpressing HEK293T cells (Figs. 1D and S1.A). These data indicated that ING4 was associated with NCL, GNL2, NOLC1 and GNL3 *in vivo*.

With purified proteins *in vitro*, glutathione S-transferase (GST)-tagged GNL3, but not GST, pulled down recombinant purified His-ING4 (Fig. 1E). NOLC1 also interacted with ING4 *in vitro* (Fig. 1F). We used a competitive assay to examine the binding of NOLC1 and GNL3 to ING4. Under the condition with a constant amount of NOLC1, addition of increasing amounts of GNL3 did not affect NOLC1 binding to the ING4 beads (Fig. S1B). The same result was obtained with the constant amount of GNL3 and the increasing amount of NOLC1 (Fig. S1C). These results indicated that these two proteins did not compete for ING4 binding.

In addition, we also found previously reported interacting partners of ING4^8,24^, including histone acetyltrasferase HBO1, p53, nuclear factor NF-kappa-B p65 subunit and histone H3 in the pull-down complex (Fig. S2).

### PHD domain was essential and sufficient for ING4 nucleolar localization

**Figure 2:**
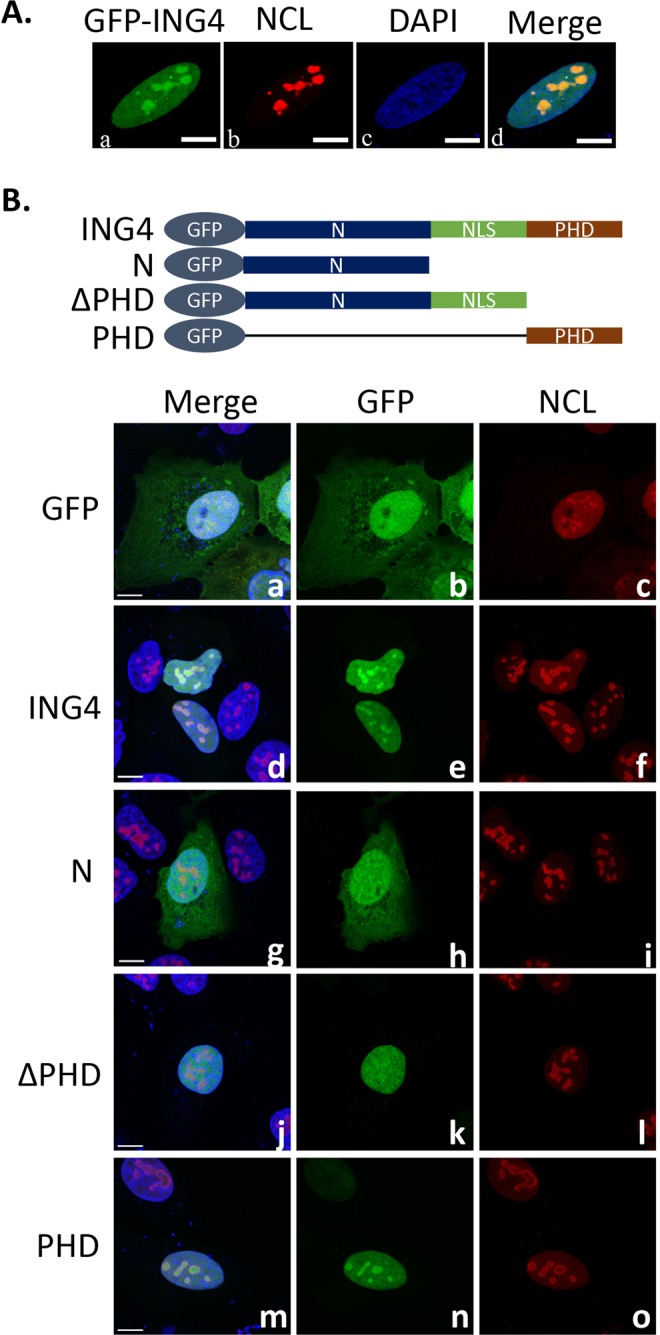
PHD domain was essential and sufficient for ING4 nucleolar localization. (**A**) Colocalization between GFP-ING4 and NCL in U-2 OS cells was evaluated by immunofluorescence study. Photos a, b, c, and d show GFP-ING4 in green, NCL in red, DAPI in blue and their merge, respectively. The data shown are the representative of three independent experiments with similar results. Scale bars in the figures indicate 10 µm. (**B**) GFP-fused ING4 domains that were used in the experiments were schematically shown. U-2 OS cells expressing various GFP-ING4 domains indicated in the photos were evaluated for GFP (green), NCL (red) and DAPI (blue). The data shown are the representative of three independent experiments with similar results. Scale bars in the figures indicate 10 µm.

We next investigated intracellular localization of ING4 by expressing GFP-ING4 in U-2 OS cells and comparing with the localization of a nucleolar marker protein, NCL^25^. GFP-ING4 was detected in the nucleus with strong accumulation in the nucleolus, but not in the cytoplasm (Fig. 2A). ING4 is comprised of the N-terminal (N) domain, the nuclear localization signal (NLS) domain and the PHD domain, which also contains another nuclear localization signal^4^. We examined which domain was critical for the nucleolar localization by generating cells expressing GFP-fused various ING4 domains as indicated in Fig. 2B (upper panel). Here, the nuclear compartment and the nucleolus were stained with DAPI and the fluorescent-conjugated antibody against NCL respectively. The NLS-containing domain (ΔPHD) localized in the nucleus although it was not condensed in the nucleolus (Fig. 2B,j–l). On the other hand, the PHD domain alone localized in the nucleus with nucleolar accumulation (Fig. 2B,m–o) like the full-length ING4 (Fig. 2B,d–f). Thus, the PHD domain was essential and sufficient for nucleolar localization of ING4.

### ING4 positively regulated cell proliferation of HAP1 cells

**Figure 3:**
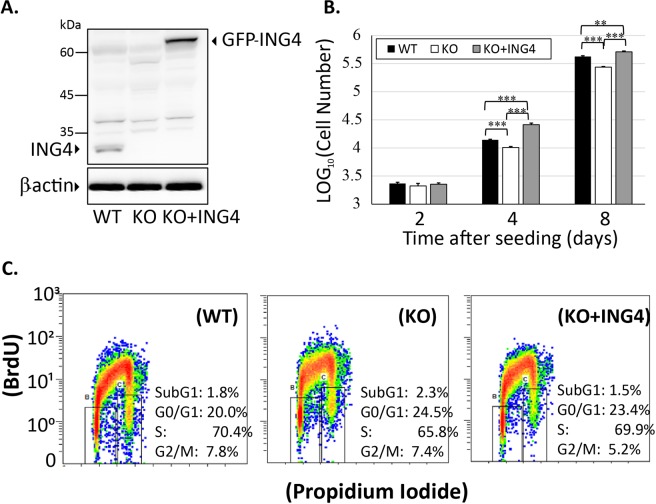
ING4 positively regulated cell proliferation of HAP1 cells. (**A**) The expression of ING4 in the various types of HAP1 cell lysates was evaluated by the western blot analysis with anti-ING4 and anti-β-actin antibodies. Lane 1: wild type HAP1 cells, Lane 2: ING4-KO HAP1 cells, and Lane 3: ING4-KO HAP1 cells that were rescued by expression of GFP-ING4. (**B**) Three types of HAP1 cells described in (**A**) were seeded in 12-well plates at a density of 4 × 10^3^ cells/well, then harvested on day 2, 4, and 8. The cell numbers were counted and displayed in the logarithm to the base 10. The data shown are means ±SD in three independent experiments. **P < 0.01, ***P < 0.001. (**C**) HAP1 WT, KO and GFP-ING4-rescued KO cells used in this study were analyzed by the flow cytometry. Cell proliferation was analyzed by labelling with anti-BrdU antibody and DNA dye Propidium Iodide (PI). The flow cytometry analysis was based on 20,000 single-cell events. B: G_0_/G_1_ population, C: G_2_/M population.

To investigate the role of ING4 in regulation of cell growth, we disrupted ING4 gene in haploid HAP1 cells using the CRISPR-Cas9 system. Since haploid HAP1 cells tend to become diploid and form a mixture of haploid and diploid cells during culture as described by the manufacturer, we established the diploid cell line by the flow cytometry screening (Fig. S3). We used wild type (WT) and ING4-deficient (KO) diploid HAP1 cells in following experiments. Then, a rescued diploid ING4-KO HAP1 cell line was established by exogenous expression of GFP-ING4. The expression level of GFP-ING4 in the rescued cells was comparable to the endogenous level in the WT cells (Fig. 3A).

We examined the proliferation velocity of the HAP1 cells. The number of ING4 KO cells was 72% of that of WT cells on day 4 (P < 0.001) and 65% (P < 0.001) on day 8 (Fig. 3B). Importantly, the reduced proliferation rate of the ING4-KO cells was rescued by expression of exogenous GFP-ING4 (Fig. 3B). We then used flow cytometry to explore whether ING4 has a role on cell cycle progression (Fig. 3C). Bromodeoxyuridine (BrdU)/propidium iodide (PI) double staining showed that the number of apoptotic cells shown in subG1 population in KO cells (2.3%) was similar to that in WT and rescued cells (1.8% and 1.5% respectively). The populations of proliferating cells (S + G2/M) were also similar: 73.2% in KO cells compared with 78.2% and 75.1% in WT and rescued cells respectively (Fig. 3C). Thus, ING4 could positively regulate the cell growth in HAP1 cells without affecting cell cycle checkpoints.

### ING4 deficiency reduced the pre-rRNA level

**Figure 4:**
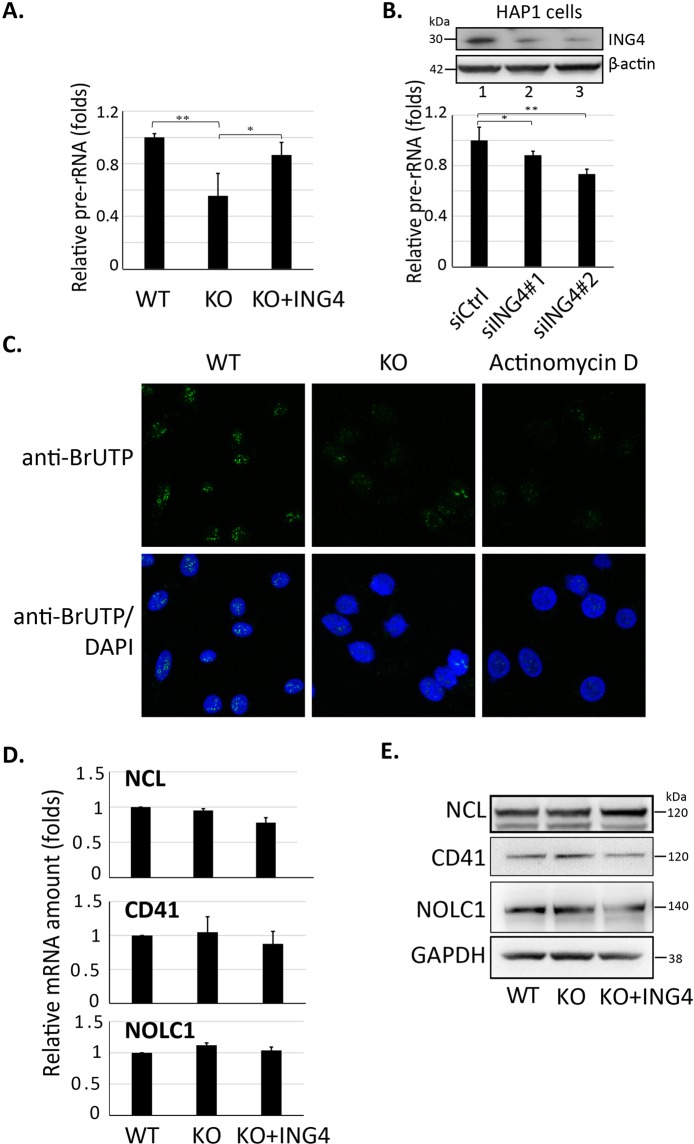
ING4 promoted the rRNA synthesis. (**A**) Equal amounts of total RNA extracted from WT, ING4-KO and GFP-ING4 rescued HAP1 cells were converted into cDNA, followed by RT-qPCR in triplicate. The relative pre-rRNA amounts were calculated by using the 2^−ΔΔCt^ method with β-actin normalization. The results shown are presented means ±SE in three independent experiments. (**B**) Similar experiments were performed with siRNA-mediated knockdown of ING4 in HAP1 cells. The upper insets display western blot results for ING4 or β-actin proteins. siCtrl: cells were transfected with negative control siRNA (Lane 1). siING4: cells were transfected with siRNA targeting ING4 at sequence #1 (Lane 2) or #2 (Lane 3). siRNA sequences were shown in Supplementary Table S1. The results shown are presented means ±SE in three independent experiments. *P < 0.05. (**C**) ING4 depletion reduced newly synthesized rRNA in HAP1 cells. HAP1 cells with/without 1-hour actinomycin D pre-treatment were incubated in a bundle of ribonucleotides including 5-BrUTP for 20 min before fixing for subsequent immunofluorescence. 5-BrUTP that incorporated into nascent rRNA was probed by Alexa Fluor 488-conjugated antibody and detected by confocal microscope. The full images were shown in Supplementary Information. (**D**,**E**) The specific consequence of ING4 depletion. ING4 expression levels had no effect on transcription (**D**) or protein (**E**) levels of several representative genes or proteins. Relative mRNA amounts were evaluated by RT-qPCR in normalization with β-actin. Protein levels were examined by western blot with specific antibodies as indicated in the figure.

Since ING4 was dense in nucleoli (Fig. 2) and interacted with several nucleolar proteins implicated in ribosome biogenesis (Fig. 1), we sought to examine the role of ING4 in the regulation of rRNA synthesis. Here, we measured the level of 47S precursor ribosomal RNA (pre-rRNA), the first product of ribosomal DNA (rDNA) transcription by the real-time quantitative PCR (RT-qPCR). We found that ING4-KO HAP1 cells exhibited the lower pre-rRNA level compared to that in the WT cells. This reduction in the pre-rRNA level was rescued by exogenous expression of GFP-ING4 (Fig. 4A). We also examined the pre-rRNA level in HAP1 and U-2 OS cells with the transient knockdown of ING4 mRNA by two different siRNAs. Both siRNAs were effectively reduced ING4 expression and pre-rRNA levels in both cell lines, compared to the negative control siRNA (Figs. 4B and S4A). We then analyzed the effect of ING4 deficiency on the synthesis of pre-rRNA by the nuclear run-on assay. We incubated U-2 OS cells with ribonucleotide mixture containing the uridine analogue 5-bromouridine 5′-triphosphate (5-BrUTP) and evaluated the newly synthesized RNA in cells (Figs. 4C and S4B). Because rRNA is synthesized at high rates exclusively in nucleoli, 5-BrU immunostaining in nucleoli reflects the newly synthesized rRNA as shown previously^26^. After 20 min of incubation with 5-BrUTP, the nascent rRNA emerged in nuclei of WT cells whereas a much lesser extent was observed in KO cells. The rRNA synthesis was inhibited completely in cells pre-treated with actinomycin D, a transcription inhibitor (Fig. 4C).

Next, we examined the specificity of ING4’s effect on rRNA synthesis by analyzing the levels of other genes. The RT-qPCR showed that the transcription level of CD41, NOLC1 or NCL was unchanged, regardless of the ING4 expression level in HAP1 cells (Fig. 4D), suggesting that the effect of ING4 on the rRNA transcription was rather specific. Furthermore, we analyzed the protein level of NCL, a cell-attachment protein CD41 and NOLC1 in HAP1 cells. We found no effect of ING4 expression level on the cellular level of these proteins.

### ING4 regulated the acetylation of H3K9 and histone H4

**Figure 5:**
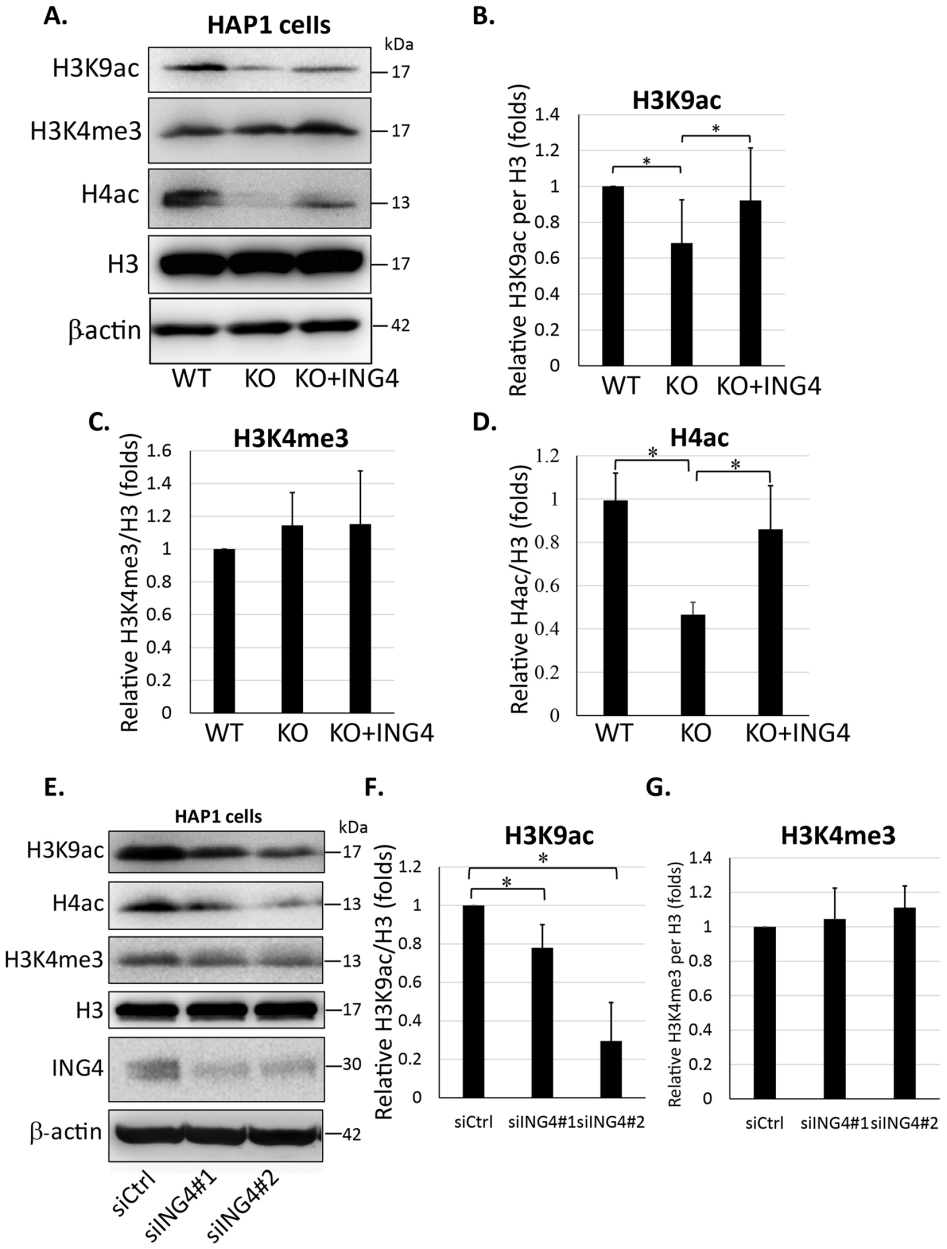
ING4 enhanced H3K9 and histone H4 acetylation. (**A**) Levels of various modified histones were detected by western blot in three types of HAP1 cells, WT: wild type cells. KO: ING4-KO cells, KO + ING4: the GFP-ING4-rescued ING4-KO cells. Total histone H3 was used as the loading control. (**B**–**D**) The relative levels of H3K9ac (**B**), H3K4me3 (**C**) and H4ac (**D**) were evaluated by the densitometric analysis with the results of (**A**). The results shown are presented means ±SD in three independent experiments. *P < 0.05. (**E**) Levels of indicated proteins were detected by western blot in siRNA-treated HAP1 cells. siCtrl: cells were transfected with negative control siRNA. siING4: cells were transfected with siRNA targeting ING4 at sequence #1 or #2. Total histone H3 was used as the loading control. (**F**,**G**) The relative levels of H3K9ac (**F**) and H3K4me3 (**G**) in the western blot performed in (**E**) were quantified in the same way above. The results shown are presented means ±SD in three independent experiments. *P < 0.05.

It has been reported that the ING proteins specifically bind trimethylated histone H3 at the 4^th^ lysine (H3K4me3), which is usually enriched at the promoter of active genes through the PHD domains. Because ING4 makes the stable complex with HBO1, a histone acetyltransferase^27–29^, and JADE, a histone H4 binding protein^1,30,31^, it is speculated that ING4 increases rRNA synthesis via regulation of histone modifications. We first evaluated the cellular levels of H3K4me3, acetylated histone H3 at the 9^th^ lysine (H3K9ac) and acetylated histone H4 (H4ac), which are known to enhance the gene transcription^30^. While the H3K4me3 level remained unchanged regardless of the states of ING4 expression, the H3K9ac level was decreased in the ING4-KO HAP1 cells compared to that in the WT cells, which was rescued by exogenous GFP-ING4 expression (Fig. 5A–C). It was also the case for H4ac in the ING4-KO cells (Fig. 5D). Transient knockdown of ING4 exhibited similar effects on the histone modifications in not only HAP1 but also U-2 OS cells (Figs. 5E–G and S5). Thus, ING4 promoted acetylation of H3K9 and H4.

### ING4 impacted the accumulations of H3K9ac and UBF at rDNA promoters

**Figure 6:**
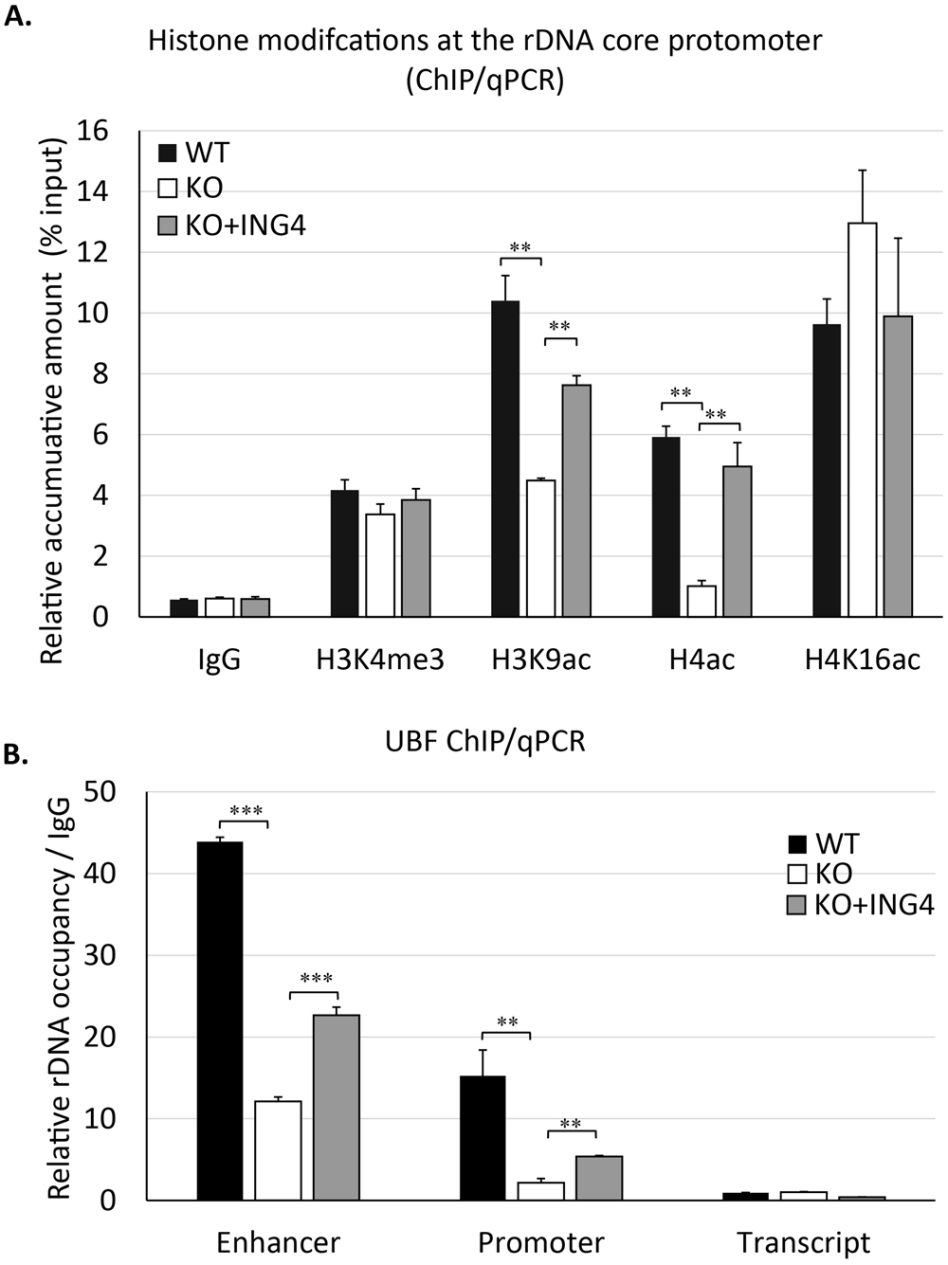
ING4 affected accumulation of histone modifications and UBF at the rDNA promoter. (**A**) The enrichment of H3K4me3, H3K9ac, H4ac and H4K16ac at the core promoter of rDNA was evaluated by ChIP/qPCR. qPCR was carried out in triplicate with chromatin DNA that was immunoprecipitated with the non-specific IgG or the specific antibodies. (**B**) The occupancy of UBF at enhancer (−1000 bp), core promoter (−50 bp) and transcript (+1000 bp) sites of rDNA was evaluated by ChIP/qPCR. It is noted that the transcription starting site is considered as +1. The primer sequences were provided in the Supplementary Table S1. The results shown are presented means ±SE in three independent experiments. **P < 0.01 and ***P < 0.001.

We next focused on the histone modifications at the core promoter region of rDNA with the established HAP1 cell lines. As demonstrated in the Fig. 6A, the chromatin-immunoprecipitation followed by RT-qPCR (ChIP/qPCR) analysis revealed that the H3K9ac level detected at the rDNA promoter was decreased in ING4-KO HAP1 cells compared to that in the WT cells. This reduction was rescued by exogenous expression of GFP-ING4 (Fig. 6A). At the rDNA promoter, the depletion of ING4 also reduced the accumulation of H4ac while it did not change the level of H4K16ac, which has been demonstrated to inhibit rRNA transcription^32,33^. It is noted that the level of H3K4me3 at the promoter region of rDNA was not affected by the level of ING4 in HAP1 cells (Fig. 6A).

We then examined the presence of UBF, the key transcription regulator of rRNA synthesis, at enhancer, core promoter and transcription regions of rDNA in the HAP1 cell lines. UBF was found to accumulate at the enhancer and the promoter but not the transcript region (Fig. 6B). ING4 depletion reduced the UBF level to a third of WT cells at both the enhancer and promoter regions of rDNA. Importantly, the reduction was recovered with exogenous expression of GFP-ING4 (Fig. 6B). Thus, ING4 depletion caused not only the alterations of histone modifications but also the reduction of UBF at the rDNA promoter.

### Nucleolar shape was altered by the loss of ING4

**Figure 7:**
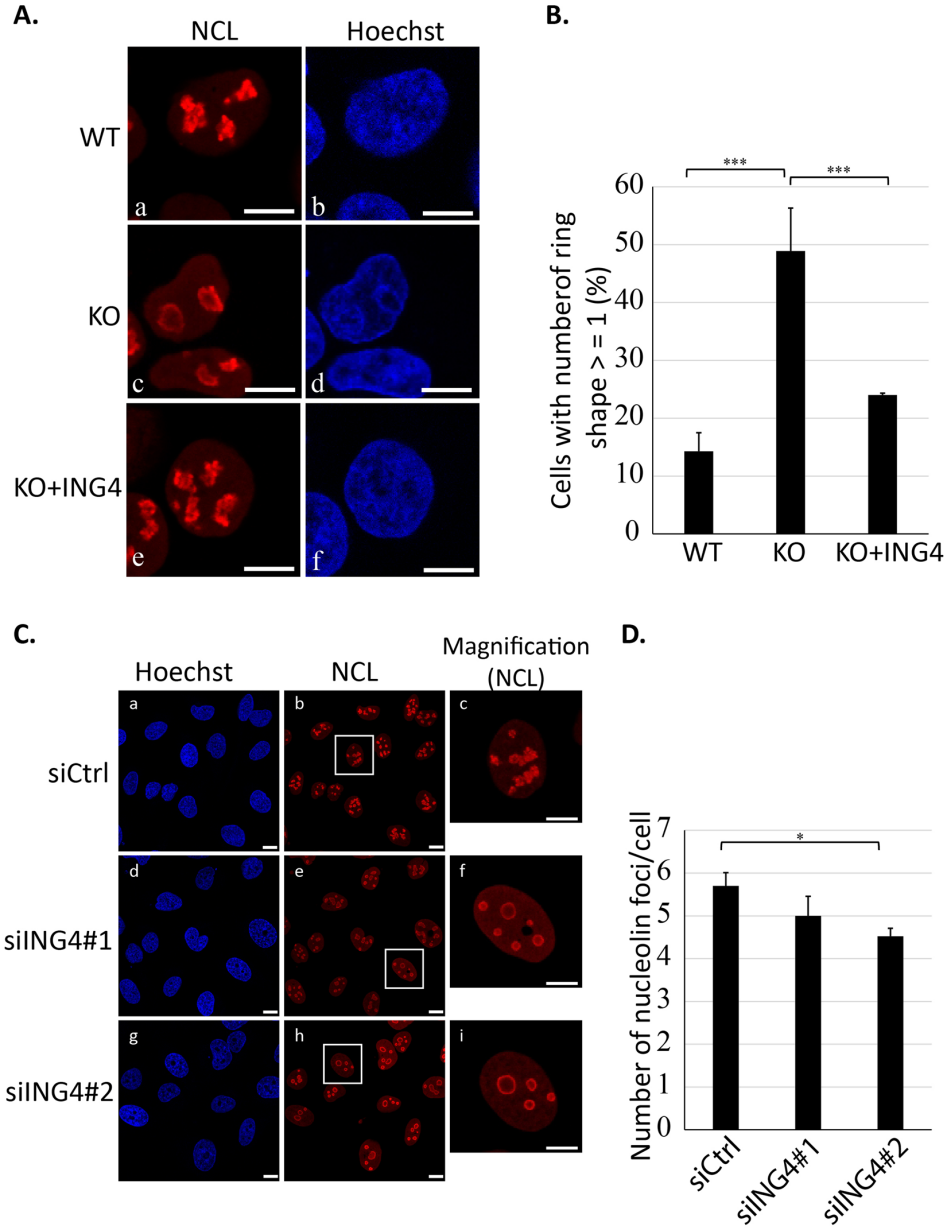
ING4 levels regulated nucleolar morphology. (**A**) The localization of NCL in wild type (WT), ING4-KO (KO) and the GFP-ING4-rescued ING4-KO HAP1 (KO + ING4) cells were evaluated by the immunofluorescence study (left photos, in red). Nuclear staining (right photos, in blue) with Hoechst was also performed. The data shown are the representative of three independent experiments with similar results. Scale bars indicate 10 µm. (**B**) The proportion of cells with at least one ring-shaped nucleolus were calculated as described in the Methods. The data shown are means ±SD in 3 independent experiments. ***P < 0.001. (**C**) ING4-knockdown U-2 OS cells and the control U-2 OS cells were generated by transfecting with ING4-specific siRNA #1 or #2, and the negative control siRNA (siCtrl). Nuclei stained with Hoechst (left photos; in blue) and NCL (middle photos; in red) are shown. The photos on the right exhibited high magnification images of inset squares from the corresponding photos in the middle, (c) was from (b), (f) was from (e), and (i) was from (h). The data shown are the representatives of three independent experiments with similar results. Scale bars indicate 10 µm. (**D**) Numbers of NCL foci per cell were counted for at least 200 cells. The data shown are presented as means ±SE in 3 independent experiments. *P < 0.05.

Nucleolar organization tightly links to the activity of ribosome biogenesis^34^. We examined the effect of ING4 depletion on nucleolar morphology by tracking localization of endogenous NCL. In ING4-KO HAP1 cells, NCL was observed at the periphery of the nucleoli to form ring-like structures, in contrast to the lobe-like structures in the WT and the GFP-ING4-rescued ING4-KO cells (Fig. 7A). We quantified the cells that contained at least one nucleolus with ring-shaped NCL localization. Approximately 50% of ING4-KO cells exhibited the abnormal nucleolin distribution, while only 15% of the WT cells and 24% of the rescued cells exhibited it (*P* < 0.001) (Fig. 7B).

The lobe-like structure detected by NCL localization was also observed in U2OS cells (Fig. 7C). In the ING4-knockdown cells, most cells showed the ring-shaped nucleoli, similar to those found in ING4-KO cells (Fig. 7C,d–i). Furthermore, ING4 knockdown significantly reduced the number of nucleoli in a cell (Fig. 7D).

## Discussion

In this study, we demonstrated that ING4 positively regulated rRNA synthesis by showing that 1) ING4 interacted with several nucleolar proteins implicated in rRNA synthesis, 2) ING4 deficiency caused the alteration of nucleolar structure, reduced the cell proliferation and reduced the rRNA transcription level that were rescued by exogenous expression of GFP-ING4, 3) ING4 deficiency also diminished the levels of H3K9 acetylation (H3K9ac) and UBF at the promoter region of rDNA.

ING4 localized in the nucleus with high nucleolar accumulation, which was consistent with previous reports^35,36^. We here demonstrated that the PHD domain alone was able to mediate the nucleolar localization (Fig. 2B). Different from other ING protein members with only nuclear localization signal in the NLS domain, ING4 possesses an extra sequence of nuclear localization at the C terminal end in the PHD domain^4^. This explained why the PHD domain alone exhibited nuclear retention (Fig. 2B). The nucleolar targeting signal is under intensive investigation and it has been shown that the common pattern is rich in R and K^37,38^. The RGKWFCPRCSQERKKK sequence at the C-terminal end of the PHD domain could lead ING4 to the nucleolus.

Furthermore, we found several nucleolar proteins as ING4-binding proteins (Fig. 1A). Then, we showed that ING4 interacted directly with GNL3 and NOLC1 both *in vitro* and *in vivo* (Fig. 1C–F). It has been known that NOLC1 facilitates rRNA synthesis through interacting with Pol I^21,39^, suggesting a role of ING4 in rRNA synthesis. Additionally, the co-immunoprecipitation experiments showed that ING4 was associated with NCL *in vivo* (Fig. 1B). NCL also plays a critical role for ribosome biogenesis, including rRNA synthesis and later processing of rRNA^25,40^. Thus, the effect of ING4 might be mediated by these proteins.

We demonstrated a correlation between pre-rRNA levels and ING4 expression levels by showing that KO or knockdown of ING4 in cells exhibited the decreased pre-rRNA level, which was rescued by the expression of exogenous GFP-ING4 (Fig. 4A,B). We found that ING4 deficiency suppressed the transcription that form nascent pre-rRNA molecules in nucleoli (Fig. 4C). The effect of ING4 on the rRNA synthesis is rather specific since the transcription and the expression levels of NOLC1, GNL3 or CD41 were not affected by the ING4 expression level (Fig. 4D,E). Although H3K4me3 would be present in the promoter site of many active genes, ING4 did not enhance the transcription level of these genes. The nucleolar interacting proteins of ING4 might play a critical role in this specificity. Further examinations are absolutely required.

Ribosome biogenesis is tightly associated with cellular activities, including cell proliferation and cell cycle progression^41–43^. Recently ING1 with its inhibitory function on cell proliferation has been reported to suppress ribosome biogenesis^44^. In this study, we clearly showed that ING4 positively regulated cell proliferation by comparison between WT and ING4-KO HAP1 cells (Fig. 3B). Among the controversial functions of ING4 on the cell proliferation, our data supported the positive role of ING4. Along with ING4, ING3 and ING5 have also been reported to enhance the proliferation of several cancerous cell lines^6,8,9^. It is noted that ING1 and ING 2 are associated with histone deacetylases (HDACs) while ING3, ING4 and ING5 are with histone acetyltransferases (HATs)^1,8^. There is a similar situation in yeasts, among three ING homologs, Yng1 and Yng2 are associated with HATs whereas Pho23 forms a complex with an HDAC^45–47^. A research on yeasts has pointed out that Pho23 inhibits p53-dependent activation of p21, whereas Yng2, an ING1 homolog, is required for p21 promoter transactivation by p53^48^. Interestingly, the opposite roles of these yeast ING proteins are dependent on their respective histone-modifying components^49^. Given the conservation between human and yeast INGs^45^, we hypothesized that the function of ING proteins in the cell proliferation also was dependent on its binding factors, HDACs or HATs.

Since ING4 forms a stable complex with HBO1, a HAT, and JADE, a histone H4 binding protein^1^, we examined whether the ING4’s function in the up-regulation of rRNA transcription was mediated by histone modifications. Interestingly, the cellular levels of H3K9ac and H4ac, which have been considered to enhance transcription^30^, were dependent on ING4 expression levels while the H3K4me3 level was not (Fig. 5). It was conceivable because ING4 bind to H3K4me3 in the promoter region of an active gene though its PHD domain^4,50^. Recently, several groups have showed that H3K4me3 and H3K9ac are strongly enriched at the promoter region of rDNA^51,52^, suggesting the important of these modifications in regulation of rRNA transcription. In our study, the level of H3K4me3 was unchanged regardless of ING4 protein levels. However, we here detected the downregulation of H3K9ac level at the promoter region of rDNA with ING4 deficiency, which was recovered by the exogenous expression of GFP-ING4 (Fig. 6A). This recovery of the H3K9ac level at the promoter region of rDNA may be mediated by the HBO1 in the ING4-containing complex. For histone H4 acetylation, JADE containing H4 binding domain may play some roles in the process, in collaboration with HBO1^1,53^. Although acetylation of histone H4 is generally linked to active chromatin^50^, acetylation at lysine 16 of histone H4 (H4K16ac) at rDNA promoters has been demonstrated to repress their activity through recruitment of the nucleolar remodeling complex (NoRC), resulting in silence of rDNA^32,33,54^. We found that the levels of ING4 in cells was irrelevant to H4K16ac accumulation at the promoter site of rDNA (Fig. 6A). This result was fit well with a previous reports that has demonstrated HBO1 as a major impact on acetylation at H4K5, H4K8 and H4K12, but not H4K16^53^. Thus, ING4 was very likely to regulate rRNA transcription positively via enhancing the histone acetylation at the rDNA promoters.

Importantly, we could demonstrate that the recruitment of UBF, the key transcription factor of rDNA, at the rDNA promoter and enhancer regions was dependent on the ING4 levels by the ChIP/qPCR assay (Fig. 6B). Previous studies has shown that UBF modulates the rRNA transcription by preferably binding to the responsive elements in core promoter and enhancer regions^55,56^. Although the mechanism of ING4-dependent recruitment of UBF to these regions was unknown, but the enhanced acetylation of histones presumably contributed to the open chromatin structure, which would promote the accessibility of UBF to this sites^57,58^.

During ribosome biogenesis, the pre-RNA synthesis and the early processing of rRNA occur at the nucleolar inner sites with the high density of transcription factors and auxiliary proteins, while the late processing and the assembly of rRNA happen at the outer part of the nucleous^23,59^. As a nucleolar protein marker, NCL normally localizes in abundance in both the inner site and outer site^22,23^. The re-location of NCL to the nucleolar outer site is reported to be associated with loss of rRNA transcription observed in certain stress conditions^40,60,61^. We here found NCL disappeared from the nucleolar center and formed the ring-shaped nucleoli after the depletion of ING4 in HAP1 and U-2 OS cells (Fig. 7). Because NCL interacted with ING4 *in vivo* (Fig. 1B), the presence of ING4 on the promoter of rDNA could facilitate the normal recruitment of NCL to the proximity of rDNA in the inner nucleolus. The role of ING4 in rRNA synthesis could be also through keeping the normal localization of NCL in the nucleolus.

In summary, we have demonstrated that ING4 positively regulated rRNA transcription. We identified several nucleolar proteins including NOLC1, GNL3 and NCL as ING4-interacting partners. It is possible that ING4 could contributed to ribosome biogenesis through regulating these proteins in addition to histone modifications. Further examinations should be required to elucidate the involvement of ING4-interacting proteins.

## Methods

### Cell culture and reagents

Anti-ING4 (C-term) polyclonal antibody was purchased from Abgent. Mouse monoclonal antibodies against histone H3, trimethylated histone H3 lysine 4 (H3K4me3) and acetylated histone H3 lysine 9 (H3K9ac) were from Takara Bio. Monoclonal anti-nucleolin and -UBF antibodies were from Santa Cruz. Anti-green fluorescent protein (GFP) polyclonal rabbit antibody was from MBL Life Science. Horseradish peroxidase-conjugated anti-mouse IgG and anti-rabbit IgG secondary antibodies were from Jackson ImmunoResearch. Chemicals were purchased from either Sigma or Wako, unless otherwise stated. Chronic myelogenous leukemia HAP1 cells (Horizon) were grown in Iscove’s Modified Dulbecco’s Medium (IMDM). Osteosarcoma U-2 OS and cervix adenocarcinoma HeLa S3 cells (both from ATTC) were cultured in Dulbecco’s Modified Eagle’s Medium (DMEM). All media were purchased from Nacalai Tesque. All the cells were cultured in the medium supplemented with 10% fetal bovine serum (FBS) (Gibco), 100 unit/ml penicillin and 100 μg/ml streptomycin (Nacalai Tesque) at 37 °C under an atmosphere of 5% CO_2_.

### Plasmids, siRNA and transfection

A full-length ING4, GNL-3 or NOLC1 cDNA was amplified by polymerase chain reaction (PCR) from human bone-marrow cDNA (Takara Bio) with the primer sets shown in the Supplementary Table S1. The sequence was inserted into the bacterial expression vector pGEX 4T-3 (Invitrogen), or mammalian expression vectors pEGFP-C1 (Clontech). Similarly, ING4 truncated mutants were also generated by PCR using primers described in the Supplementary Table S1 and inserted into pEGFP-C1. A lentivirus expressing ING4 was generated by inserting the full-length ING4 cDNA into a lentivirus vector as described previously^62^. All the sequences after PCR were confirmed with an Applied Biosystems 3130 Genetic Analyzer system.

Pre-designed siRNAs targeting against ING4 (Thermo Fisher Scientific) were used in knockdown experiments. For transfection, 20 nM of either ING4 or Ambion® Silencer® Negative Control siRNA (Thermo Fisher Scientific) was transfected into cells with lipofectamine RNAimax (Invitrogen) accordingly to the manufacturer’s protocols. After 72 h of incubation, cells were harvested. The lysate was prepared with RIPA buffer that contained 10 mM Tris-HCl, pH 7.4, 140 mM NaCl, 1% Triton X-100, 0.1% sodium deoxycholate, 0.1% sodium dodecyl sulfate (SDS) supplemented with EDTA-free protease inhibitor cocktail (Roche). Protein concentration was monitored by Bradford method using bovine serum albumin as a control.

### Protein purification and GST affinity assay

GST-fused ING4 was purified from cytosolic fraction of overexpressing E. coli strain BL21 (DE3, from Nippon Genetics) by Glutathione Sapharose 4B (GE Healthcare) according to the manufacturer’s instruction. After the affinity purification, the eluate was further purified by an anion exchange chromatography with Mono Q column (GE Healthcare) followed by another gel filtration through a Superdex 200 (GE Healthcare) column with a buffer containing 20 mM Hepes-KOH pH 7.4, 500 mM KCl, 1 mM dithiothreitol (DTT) using AKTA system (GE Healthcare). ING4-containing fractions were collected and concentrated by centrifugal filter 30,000 NMWL (Millipore). Purified GST-ING4 was stored at −80 °C until use.

HeLa S3 cells were cultured in suspension condition. Cell lysate was prepared by incubating 1 ml of HeLa S3 pellet in 10 ml ice-cold Lysis Buffer A (20 mM Hepes-KOH pH 7.4, 100 mM KCl, 20 mM NaCl, 1% NP-40, 1 mM DTT) supplemented with EDTA-free protease inhibitor cocktail, for 10 min on ice. After sonication, a centrifugation at 20,000 × *g* for 20 min at 4 °C was performed. The supernatant was kept as the cell lysate at −80 °C until use.

GST-ING4 (5 μg) or GST (1.7 μg) was mixed with 3 ml of the cell lysate (4 mg/ml of protein concentration) and incubated overnight with rotating at 4 °C followed by another 1 h at 4 °C after adding 50 μl of Glutathione Sepharose 4B beads. The beads were then washed three times with the lysis buffer supplemented with 1 mM phenylmethanesulfonylfluoride (PMSF), followed by an elution with 100 μl of the SDS-containing sample buffer with 1% beta-mercaptoethanol. The eluates were analyzed with SDS-polyacrilmide gel electrophoresis (PAGE) followed by silver staining. Candidate proteins were determined using nanoLC/MS/MS system (DiNa HPLC system KYA TECH Corporation/QSTAR XL Applied Biosystem) from Japan Proteomics Co., Ltd (Sendai, Japan).

### Immunoprecipitation

HAP1 cells (1 × 10^7^) that stably expressed either GFP or GFP-ING4 were incubated in 1 ml of lysis Buffer B containing 50 mM Hepes (pH 7.4), 140 mM NaCl, 1% Triton X-100, 0.5% NP-40, 1 mM DTT supplemented with complete protease inhibitor cocktail, 10 nM NaF, 1 mM sodium orthovanadate, 2 mM sodium pyrophosphate and 2 mM beta-glycerophosphate. The lysates were incubated with 1.5 μg of either specific anti-GFP antibody or IgG (Sigma Aldrich) at 4 °C overnight, followed by incubation with protein G sepharose (GE Healthcare) for 3 h at 4 °C. The beads were washed 4 times in lysis Buffer B and eluted with the SDS-sample buffer with 1% beta-mercaptoethanol. The eluates were analyzed by western blot.

### Flow cytometry

HAP1 cells were pre-incubated in 30 µM BrdU (Sigma-Aldrich) for 30 min and harvested by trypsinization and washed twice with cold phosphate-buffered saline (PBS). About 1 × 10^6^ cells were fixed with cold 70% ethanol overnight. After centrifugation at 2000 × *g* for 5 min, cells were incubated in 0.5 ml 2 N HCl/0.5% Triton X-100 for 30 min, followed by a centrifugation for the pellet. After re-suspension with 0.1 M sodium tetraborate for 2 min, cells were incubated with Alexa488-conjugated antibody against BrdU (Invitrogen) for 1 h at RT. Next, the samples were washed once with PBS, followed by incubation with 10 µg/ml PI and 50 µg/ml RNase in 500 µl PBS for 30 min at room temperature. Samples were then analyzed by flow cytometry FC-500 system (Beckman Coulter). A data set collected for 20,000 cells.

### Generation of ING4 knockout (KO) and GFP-ING4 rescued HAP1 cells

ING4-KO HAP1 cells were produced by the CRISPR-Cas9 system^63^. A 20-nucleotide single guide RNA (5′-GGCACTACTCATAT-ACTCAG-3′) targeting to the second exon of ING4 gene was inserted into plasmid pSpCas9(BB)-2A-Puro (PX459) (a gift from Dr. Feng Zhang; Addgene plasmid # 48139). The plasmid was transfected into haploid HAP1 cells (Fig. S3 (i)) with Fugene6 reagent (Promega) and incubated for 24 h. After a 2-day selection with 2 µg/ml puromycin, survival cells were diluted and seeded into 96-well plate at a density of 1 cell/well. After 10 days of incubation, colonies were screened by western blot with anti-ING4 antibody to identify the presence/absence of ING4. Cell colonies without ING4 expression were further confirmed with ING4 gene specific sequencing. Although HAP1 cells were originally haploid, some of them became diploid, as shown in Fig. S3 (ii). Therefore, diploid cell colonies were screened and established by using PI staining and flow cytometry assay as described above. All the further experiments with HAP1 cells in this study were with diploid cells (Fig. S3 (iii)). GFP-ING4-rescused diploid HAP1 cells were generated by infecting ING4-KO diploid cells with the ING4 lentivirus followed by selection with blasticidin.

### RNA isolation and evaluation of rRNA transcription

Total RNA was isolated from cultured cells using Isospin Tissue&Cell RNA extraction kit (Nippon Genetics, Japan) according to the manufacturer’s instructions. The RNA was then reverse transcribed with ReverTra Ace qPCR RT Master Mix (TOYOBO). Real-time PCR was carried out in triplicate with TB Green Premix Extaq II kit (Takara Bio) on StepOne real-time PCR system (Applied Biosystems). The data were calculated based on 2^−ΔΔCt^ method^64^. rRNA transcription was evaluated by the levels of 47S precursor rRNA (pre-RNA) as described previously^40,44,65,66^. The primer set for pre-rRNA real-time qPCR was shown in the Supplementary Table S1.

### Nuclear run-on and nascent rRNA labeling

The experiment was performed as described previously^67^ with adaptation. U-2 OS or HAP1 cells were plated on glass-bottom chamber for 40 h. For negative control, the cells were pre-incubated in full medium containing 10 nM Actinomycin D. The cells were washed twice with warm PBS followed by permeabilization with P1 buffer (20 mM Tris-HCl at pH7.4, 5 mM MgCl_2_, 0.5 mM EGTA, 0.5 mM PMSF, 0.1% Triton X-100) for 10 min. The cells were then incubated in a run-on buffer (50 mM Tris-HCl at pH7.4, 100 mM KCl, 5 mM MgCl2, 0.5 mM EGTA, 20 U/ml RNasin, 1 mM PMSF, 0.5 mM each of ATP, CTP and GTP, 0.2 mM 5-BrUTP) for 20 min at 37 °C. The reaction was stopped by washing in PBS followed by fixation in 4% paraformaldehyde for 10 min and subsequent permeabilization in 0.1% Triton X-100. After blocking in 0.5% BSA in PBS for 30 min, cells were incubated with Alexa488-conjugated antibody against BrUTP for 2 h at 37 °C. The samples were observed with confocal laser scanning microscope Leica TCS SP8 (Leica Microsystem).

### Chromatin immunoprecipitation (ChIP) assay

The ChIP assay was conducted as described previously^68^ with some modifications. Briefly, 8 × 10^7^ HAP1 cells were fixed with 1% formaldehyde for 15 min at room temperature. The cross-linking reaction was quenched with 125 mM glycine for 5 min at room temperature. After a centrifugation at 500 × *g* for 5 min, the cell pellet was washed twice with cold PBS, and then suspended in 1 ml of the lysis Buffer C (50 mM Hepes KOH pH 7.4, 150 mM NaCl, 0.5% NP-40, 0.5% Triton X-100, 2 mM MgCl_2_, 1 mM DTT and protease inhibitor cocktail) and kept on ice for 10 min. Chromatin was released by treatment of 1 × 10^3^ gel units of micrococcal nuclease (NEB) for 10 min at 37 °C followed with a brief sonication. After centrifugation, 50 µl of supernatant was set aside for input. Subsequently, immunoprecipitation was conducted by incubating 50 µg chromatin with 1.5 µg antibody against either histone H3, H3K4me3, H3K9ac, UBF, H4ac or H4K16ac, or 1.5 µg IgG overnight. To reverse the crosslinking, the solutions were incubated with 0.2 M NaCl at 65 °C for 6 h. DNA was purified by the phenol/chloroform/isoamyl extraction. Subsequently, real time quantitative PCR was conducted in triplicate as described above. The histone modifications after normalization with histone H3 were evaluated based on percentage of the input. The primers were shown in Supplementary Table S1.

### Statistical analysis

Data shown in this study were expressed as the means ± SD or ±SE from three independent experiments. *P* values were calculated using two-tailed Student’s *t*-test or one-way Analysis of Variance (ANOVA) followed by Dunnett test for three-group comparisons. Differences were significant for *P* < 0.05.

## Supplementary information

Supplementary Information
