# Supplementary material for: Inhibitor of Growth 4 (ING4) is a positive regulator of rRNA synthesis
Source: Sci Rep. 2019 Nov 21;9:17235. doi: 10.1038/s41598-019-53767-1 (PMC6872537; doi:10.1038/s41598-019-53767-1)
Supplement: Supplementary file 1 — Supplementary Information [file 41598_2019_53767_MOESM1_ESM.pdf]

Inhibitor of Growth 4 (ING4) is a positive regulator of rRNA synthesis

Duc-Anh Trinh[1, 2], Ryutaro Shirakawa[2], Tomohiro Kimura[2, #], Natsumi Sakata[2], Kota Goto[2], Hisanori Horiuchi[1, 2, *]

1, Department of Oral Cancer Therapeutics, Graduate School of Dentistry, Tohoku University
2, Department of Molecular and Cellular Biology, Institute of Development, Aging and Cancer, Tohoku University

#; Present address; Research Center for Molecular Genetics, Institute for Promotion of Medical Science Research, Yamagata University Faculty of Medicine, Yamagata, Yamagata, Japan

*; To whom correspondence should be addressed; Hisanori Horiuchi, Department of Molecular and Cellular Biology, Institute of Development, Aging and Cancer, Tohoku University, Sendai, 980-8575, Japan. Tel&Fax:+81-22-717-8463, e-mail: hisanori.horiuchi.e8@tohoku.ac.jp

Materials and Methods

Protein competition assay

His-ING4 beads were generated by incubating recombinant His-ING4 with Ni-NTA agarose (QIAGEN) for 1 h in the binding buffer containing 20 mM Hepes (pH 7.4), 20 mM NaCl, 100 mM KCl, 1% Triton X-100, 1 mM DTT and 10 mM imidazole. The beads were ready after washing twice with the binding buffer. In the competition assay, a series of the mixtures between a constant amount of GST-NOLC1 and varied amounts of GST-GNL3, or vice versa, were added and mixed with the beads. After the 4-time washing with the binding buffer, bead-associated proteins were eluted with SDS-loading buffer and finally analyzed with western blot.

Figure S1. Nucleolar interactions of ING4. (**A**) ING4 interacted with GNL2 *in vivo*. Here, the lysate from HEK293T cells that temporarily expressed Flag or Flag-ING4 was incubated with anti-Flag antibody and immobilized on protein G sepharose beads. Bead-associated proteins were analyzed by the western blot with anti-GNL2 antibody (**B**) Competition assay. His-ING4 beads were generated by incubating 1 μg of His-ING4 (30 pmol) and Ni-NTA agarose beads. After washing, the beads were incubated with the mixtures that made of 10 μg of GST-NOLC1 (equal to 2-fold in mole compared to His-ING4) and different amount of GST-GNL3. The ratios of GST-GNL3/GST-NOLC1 (mole/mole) were indicated in the figure. At last, the binding proteins were analyzed by western blot with anti-GNL3 antibody for GST-GNL3 and anti-His for His-ING4. (**C**) The similar assay to (B) was conducted with the constant amount of GST-GNL3 and varied amount of GST-NOLC1.

Figure S2. GST-ING4 pull-down assay. The beads covered with recombinant GST-ING4 or GST was incubated with the cell lysate as described in the Methods. The total cell extract was used as the input in a western blot along with the above elutes. The presence of endogenous proteins were detected by specific antibodies as indicated in the figure.

Figure S3. Generation of ING4-KO and ING4-rescued KO diploid HAP1 cells. Cells were cultured in 12-well plate for 48 h before harvest by trypsinization. About $1x10^6$ cells were used in one analysis. DNA contents were evaluated with the flow cytometry after cells were stained with PI. HAP1 cells were originally haploid (i). During cell culture, HAP1 cells became mixture of haploid and diploid (ii). Only diploid cell lines were selected and established for further experiments (iii).

Figure S4. ING4 knockdown reduced rRNA synthesis. (**A**) Knockdown of ING4 in U-2 OS cells reduced the pre-rRNA pool. U-2 OS cells were transfected with 20 μM siRNA for 48 h before harvesting for protein or total RNA extraction. The upper insets display w blot results that specifically probe ING4 or β-actin proteins that were loaded in parallel in SDS-PAGE. siCtrl: cells were transfected with negative control siRNA (Lane 1). siING4: cells were transfected with siRNA targeting ING4 at sequence #1 (Lane 2) or #2 (Lane 3). siRNA sequences were shown in Supplementary Table S1. Total RNA from above cells were objects for RT-qPCR experiment whose results with β-actin normalization were presented at the lower pannel. The results shown are presented means ±SE in three independent experiments. *$*P<0.05$*. (**B**) ING4 knockdown suppressed rRNA transcription. U-2 OS cells were transfected and cultured in a glass-bottom chamber for 48 h. For the negative control, cells were pre-incubated with 10 nM actinomycin D for 3 h before permeabilizing. After 20 min of nuclear run-on, cells were fixed and newly synthesized pre-rRNA was probed with anti-BrUTP antibody. The full-size images can be seen in the Supplementary Information.

Figure S5. ING4 knockdown altered histone modification levels in U-2 OS cells. (**A**) siRNA-mediated ING4 knockdown reduced acetylation levels at H3K9 and H4 in U-2 OS cells. siCtrl: cells were transfected with negative control siRNA. siING4: cells were transfected with

siRNA targeting ING4 at sequence #1 or #2. The data shown were the preventative of western blot for each protein in three independent experiments with similar results. (**B, C**) The levels of H3K9ac and H3K4me3 respectively from (A) were quantified using ImageJ. The data are expressed as means ±SD in 3 independent experiments. *$P<0.05$*, **$P < 0.01$.

Supplementary Table S1. Primers and sequences used in this study

Figure S1

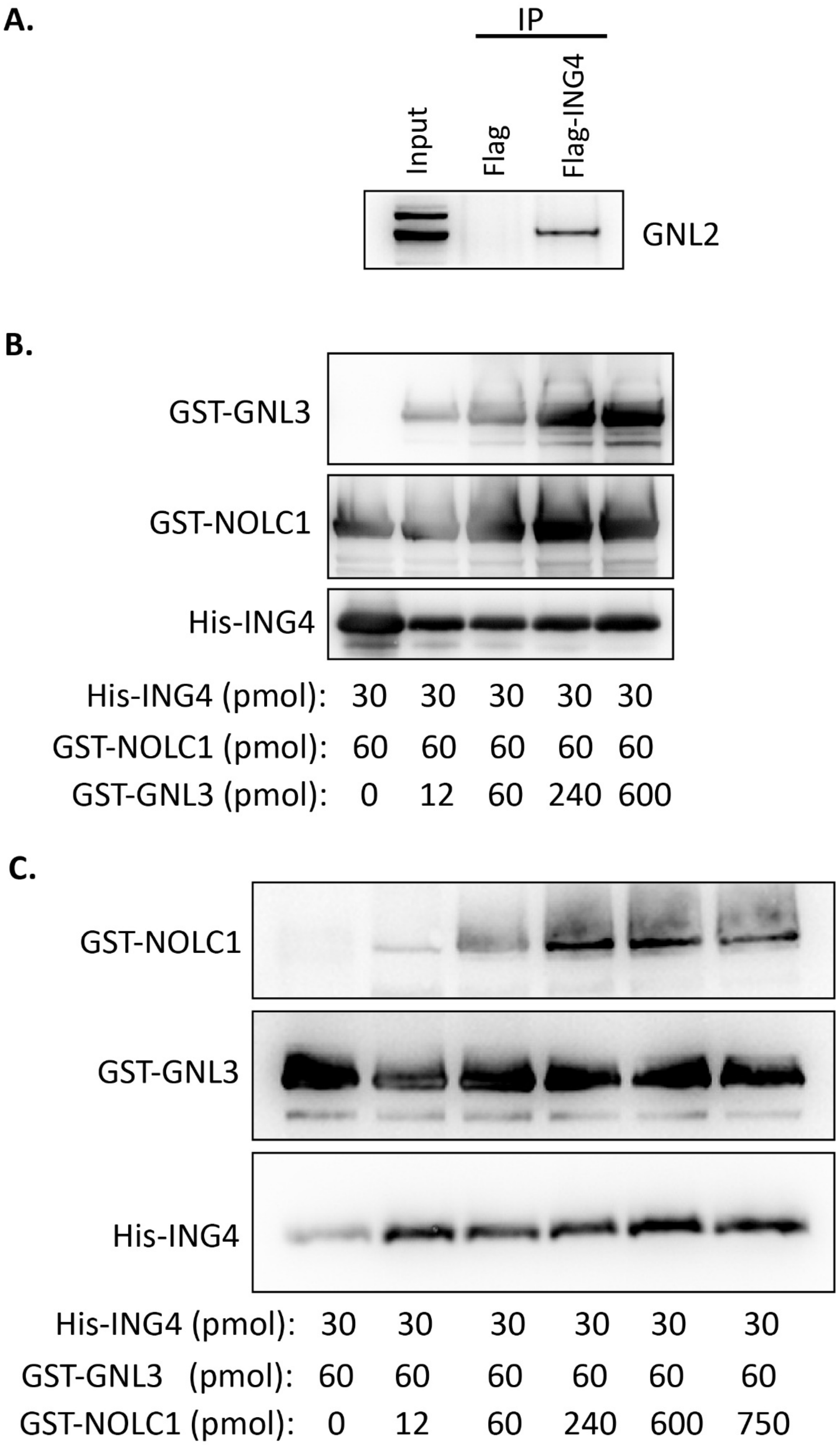
A.
IP
Input
Flag
Flag-ING4
GNL2
B.
GST-GNL3
GST-NOLC1
His-ING4
His-ING4 (pmol): 30 30 30 30 30
GST-NOLC1 (pmol): 60 60 60 60 60
GST-GNL3 (pmol): 0 12 60 240 600
C.
GST-NOLC1
GST-GNL3
His-ING4
His-ING4 (pmol): 30 30 30 30 30 30
GST-GNL3 (pmol): 60 60 60 60 60 60
GST-NOLC1 (pmol): 0 12 60 240 600 750

Figure S2

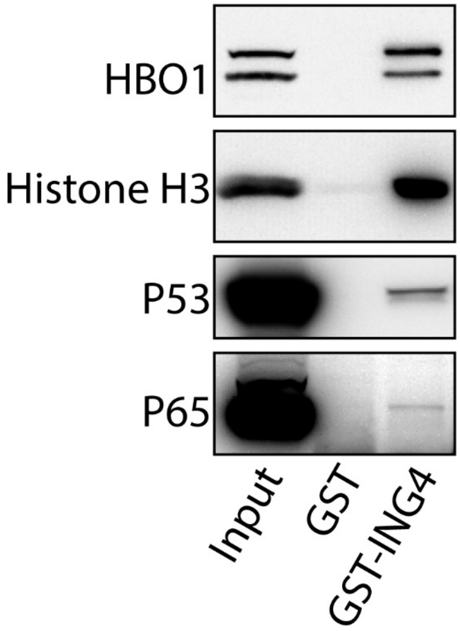

Figure S3

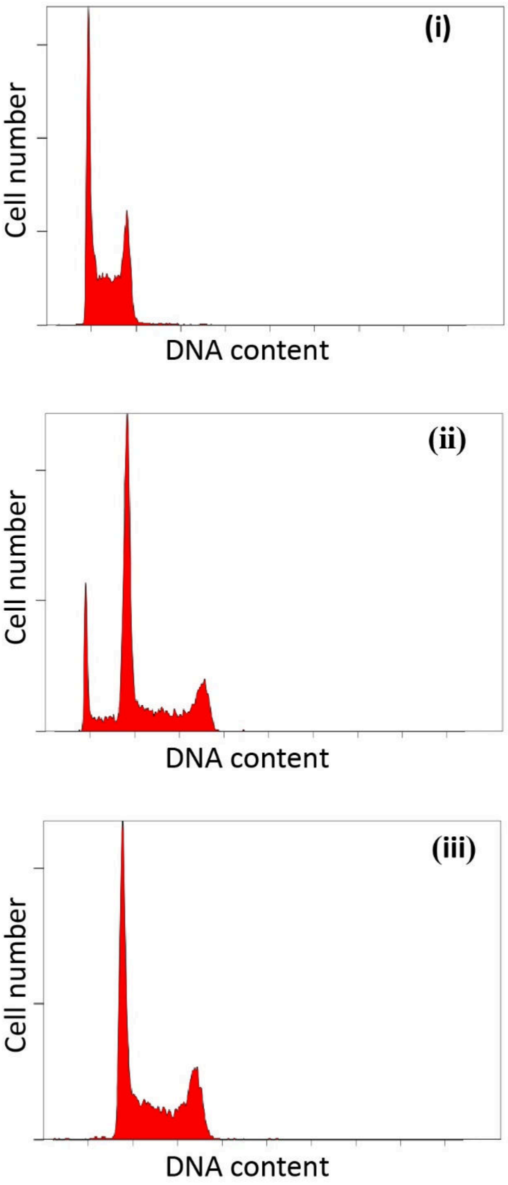

(i)
Cell number
DNA content
(ii)
Cell number
DNA content
(iii)
Cell number
DNA content

Figure S4

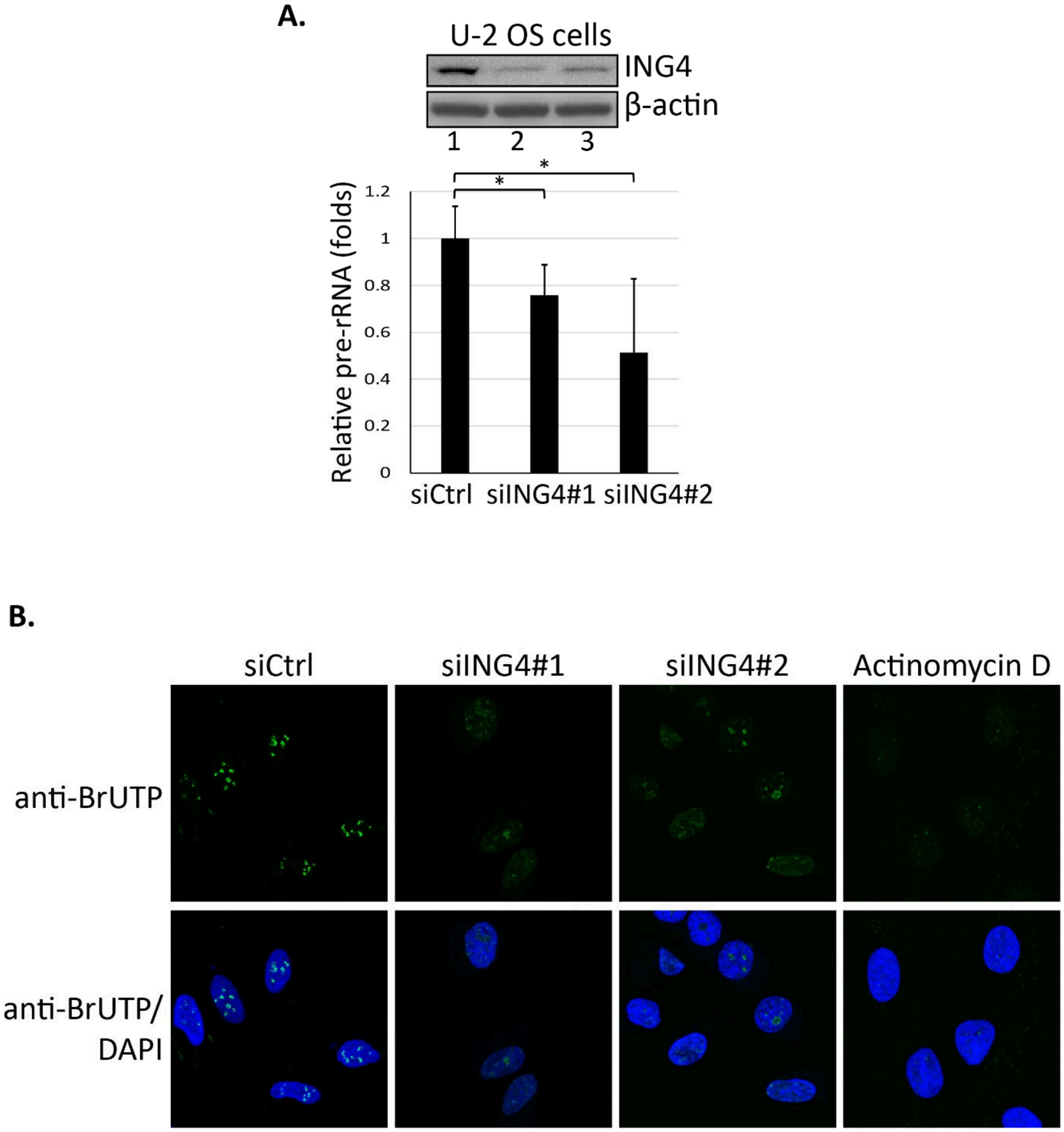
A.
U-2 OS cells
ING4
β-actin
1
2
3
*
*
Relative pre-rRNA (folds)
1.2
1
0.8
0.6
0.4
0.2
0
siCtrl
siING4#1
siING4#2
B.
siCtrl
siING4#1
siING4#2
Actinomycin D
anti-BrUTP
anti-BrUTP/
DAPI

Figure S5

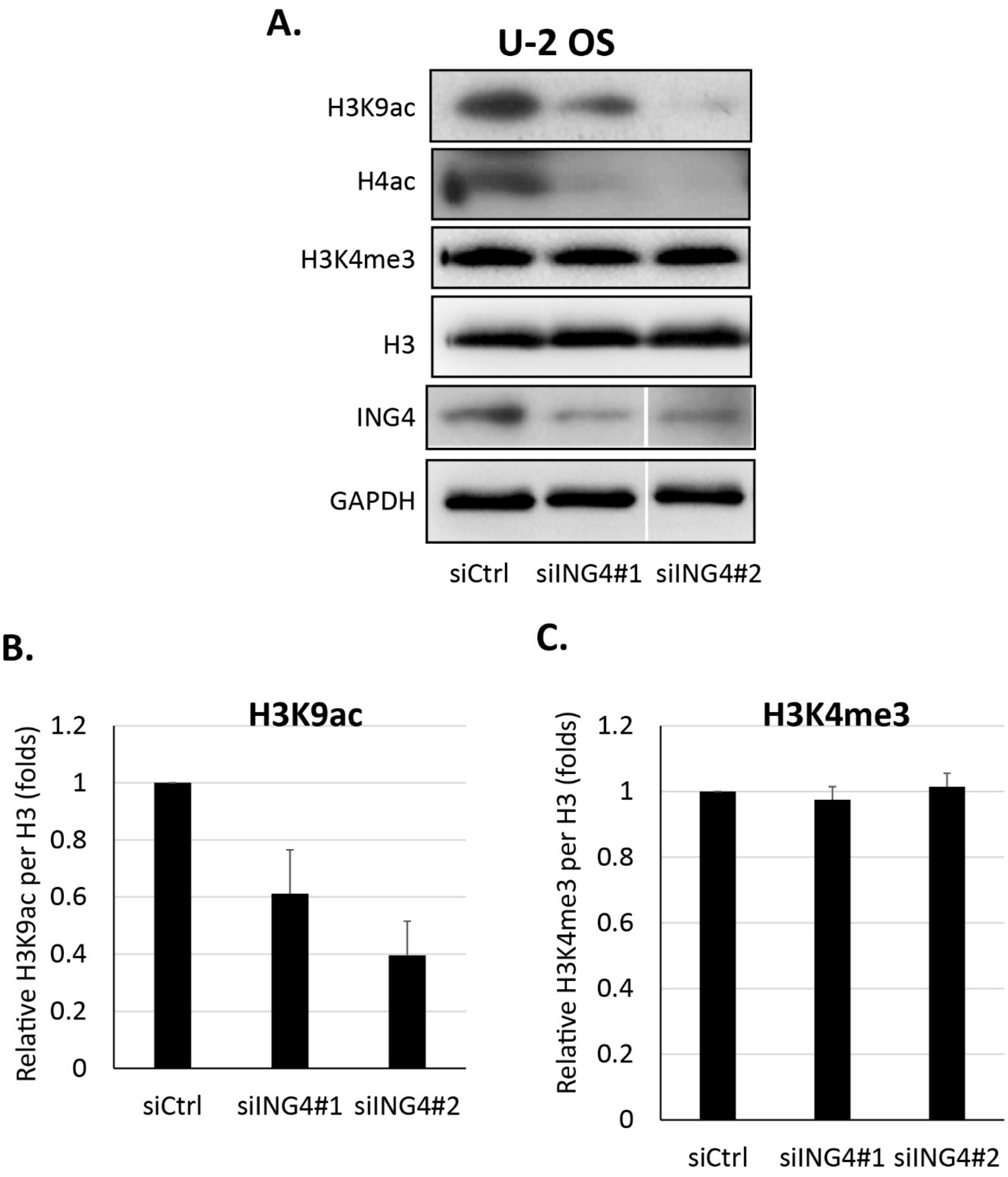
A.
U-2 OS
H3K9ac
H4ac
H3K4me3
H3
ING4
GAPDH
siCtrl
siING4#1
siING4#2
B.
H3K9ac
Relative H3K9ac per H3 (folds)
1.2
1
0.8
0.6
0.4
0.2
0
siCtrl
siING4#1
siING4#2
C.
H3K4me3
Relative H3K4me3 per H3 (folds)
1.2
1
0.8
0.6
0.4
0.2
0
siCtrl
siING4#1
siING4#2

Supplementary Table S1. Primers and sequences used in this study

Primers	Sequence
For generating full-length ING4	Forward: 5'-CATCTCGAGAGATGGCTGCGGGGATGTAT-3'<br>Reverse: 5'- AATCTCGAGCTATTTCTTCTTCCGTTCTTG-3'
For generating N fragment	Forward: 5'-CATCTCGAGAGATGGCTGCGGGGATGTAT-3'<br>Reverse: 5'- AATCTCGAGCTAGTCACTTGACTCAATC -3'
For generating ΔPHD fragment	Forward: 5'-CATCTCGAGAGATGGCTGCGGGGATGTAT-3'<br>Reverse: 5'- AATCTCGAGCTAATCCAACACATCAGAGGG-3'
For generating PHD fragment	Forward: 5'-AATGAATTCGGATATGCCTGTGGATC-3'<br>Reverse: 5'- AATCTCGAGCTATTTCTTCTTCCGTTCTTG-3'
For core promoter in ChIP/qPCR	Forward: 5'-CCCGGGGGAGGTATATCTTT-3'<br>Reverse: 5'-CCAACCTCTCCGACGACA-3'
For enhancer site in ChIP/qPCR	Forward: 5'-AGAGGGGCTGCGTTTTCGGCC-3'<br>Reverse: 5'- CGAGACAGATCCGGCTGGCAG-3'
For transcript site in ChIP/qPCR	Forward: 5'-GGCGGTTTGAGTGAGACGAGA-3'<br>Reverse: 5'-ACGTGCGCTCACCGAGAGCAG-3'
For pre-rRNA in RT-qPCR	Forward: 5'-TGTCAGGCGTTCTCGTCTC-3'<br>Reverse: 5'-AGCACGACGTCACCACATC-3'
siING4#1<br>siING4#2	5'- CAAUCAGGGUUGUCACAGC -3'<br>5'- CGAACCCACCUAUUGCCUU -3

Raw data for Figure 1A

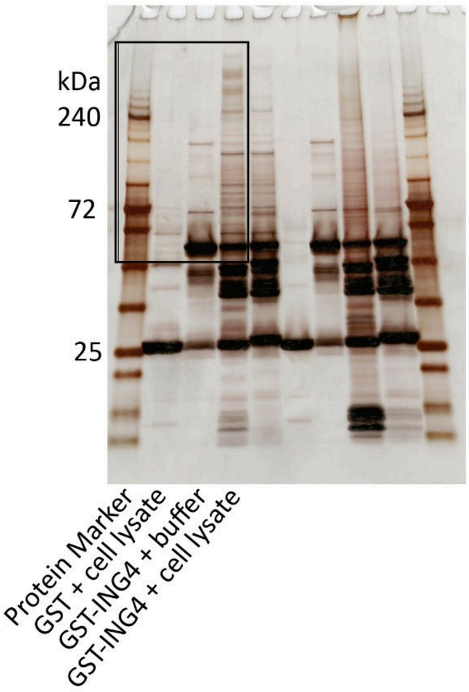

Raw data for Figure 1B

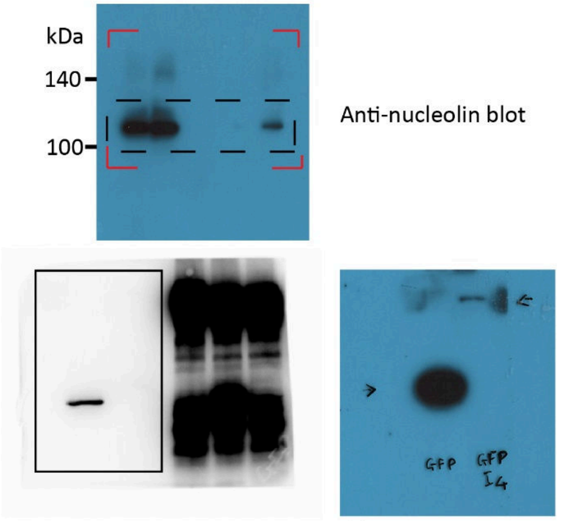

Anti-GFP blot

Note: The Fig. 1B is a crop along the dashed rectangle in the upper image. The lower part was cut for anti-GFP antibody probe as shown in two lower images. The right image was from the rectanged part with longer expose. The arrows indicate specific bands dectected by anti-GFP antibody.

Note: The Figure 1A is cropped along the black square.

Raw data for Figure 1C

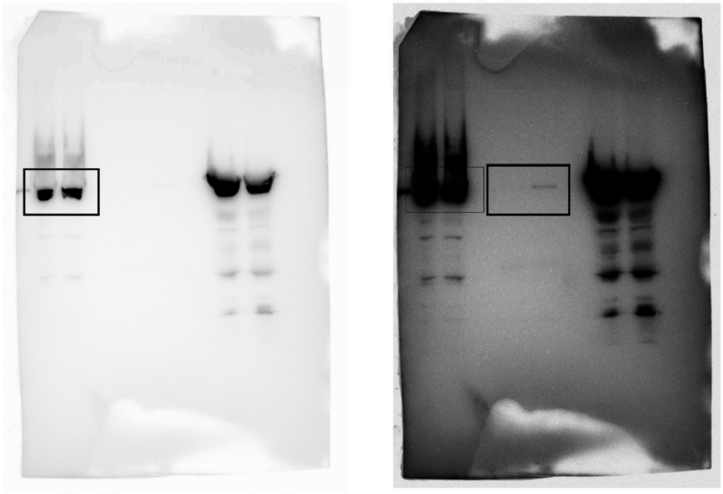

Note: The Figure 1C are cropped along the black squares from the image of short (left) or long (right) exposure

Raw data for Figure 1D-F:

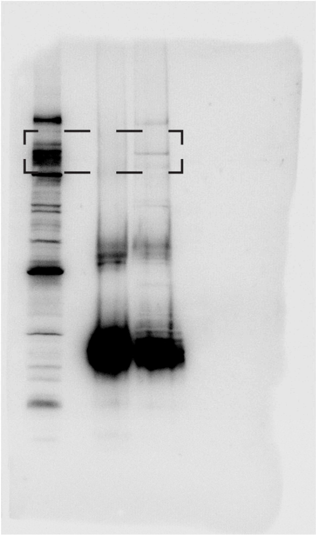

Fig. 1D. NOLC1

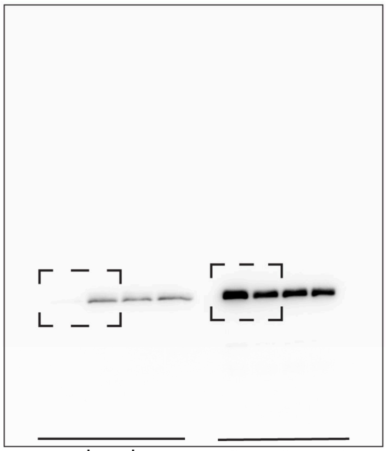

bead associated   flow-through

Fig. 1E. His-ING4 (shorter expose)

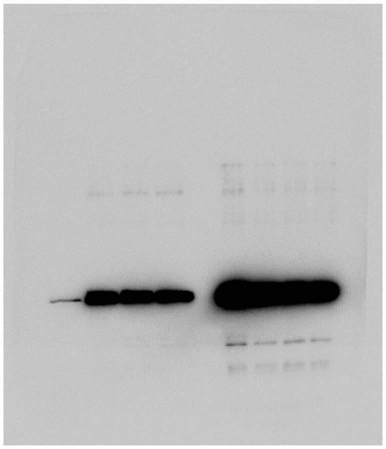

Fig. 1E. His-ING4 (longer expose)

Fig. 1f. His-ING4 (shorter expose)

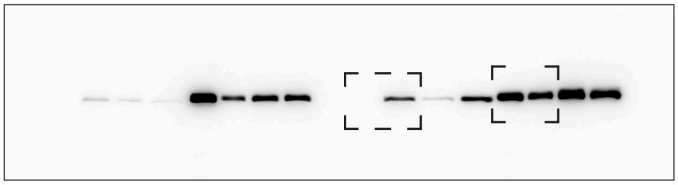

Fig. 1f. His-ING4 (longer expose)

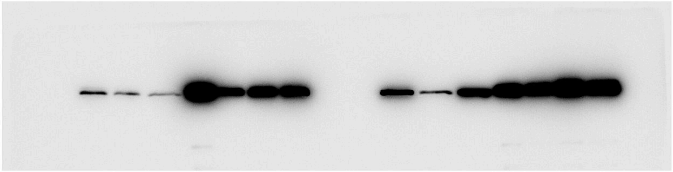

bead associated   flow-through

Note: The figures were cropped along the dashed rectangles.

Raw data for Fig. 3A

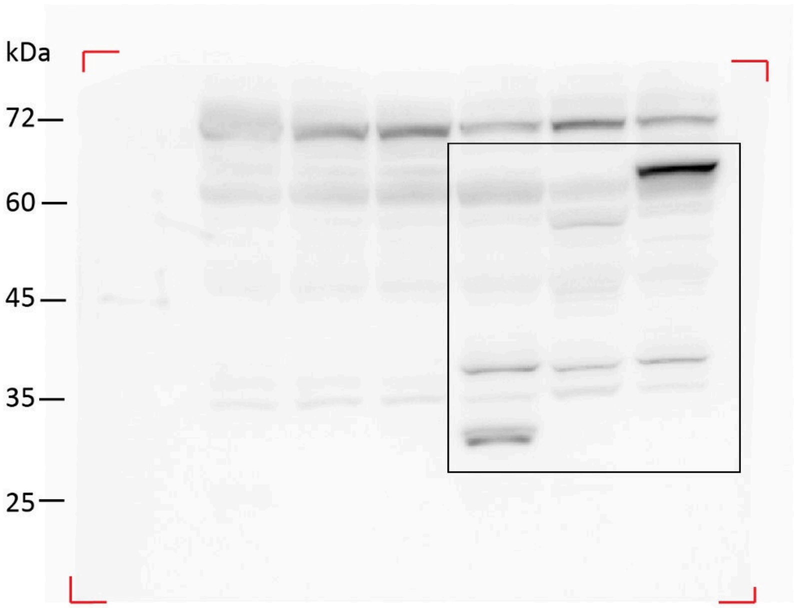

Anti-ING4 blot

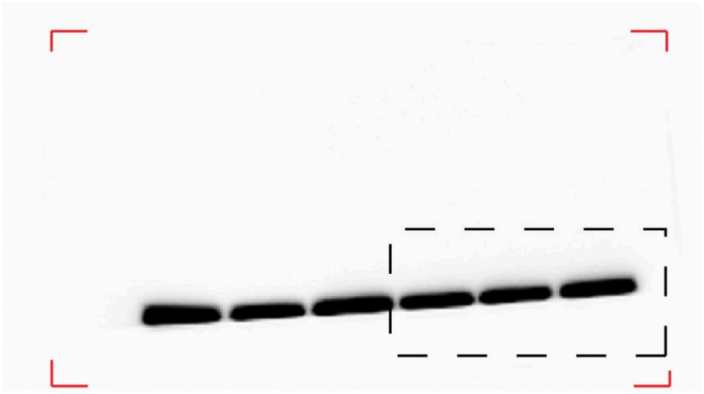

Anti-β-actin blot

Note: Blots were from the same amount of samples and conducted in the same conditions of western blot. The figures were cropped along the rectangle or dashed rectangle. The red corners indicate boundaries of the blots.

Raw blots for Figure 4

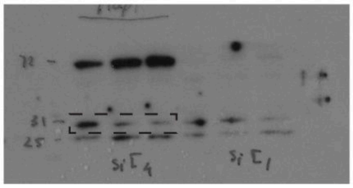

Fig. 4B. ING4

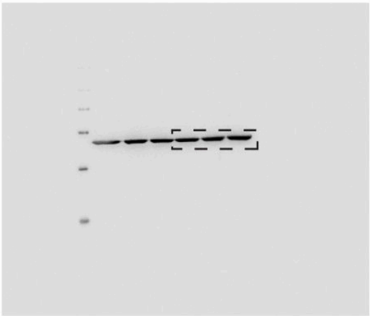

Fig. 4B. Beta-actin

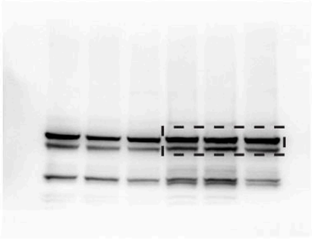

Fig. 4E. NCL

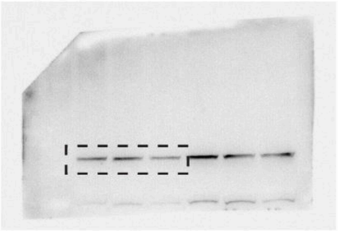

Fig. 4E. CD41

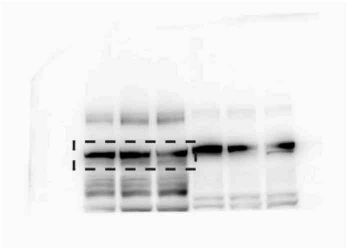

Fig. 4E. NOLC1

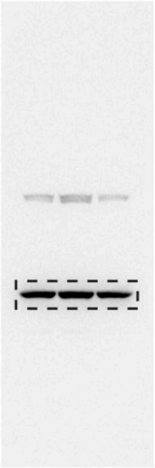

Fig. 4E. GAPDH

Note: Blots were from the same amount of samples and conducted in the same conditions of western blot. The figures were cropped along the rectangle.

Raw data for Figure 4C

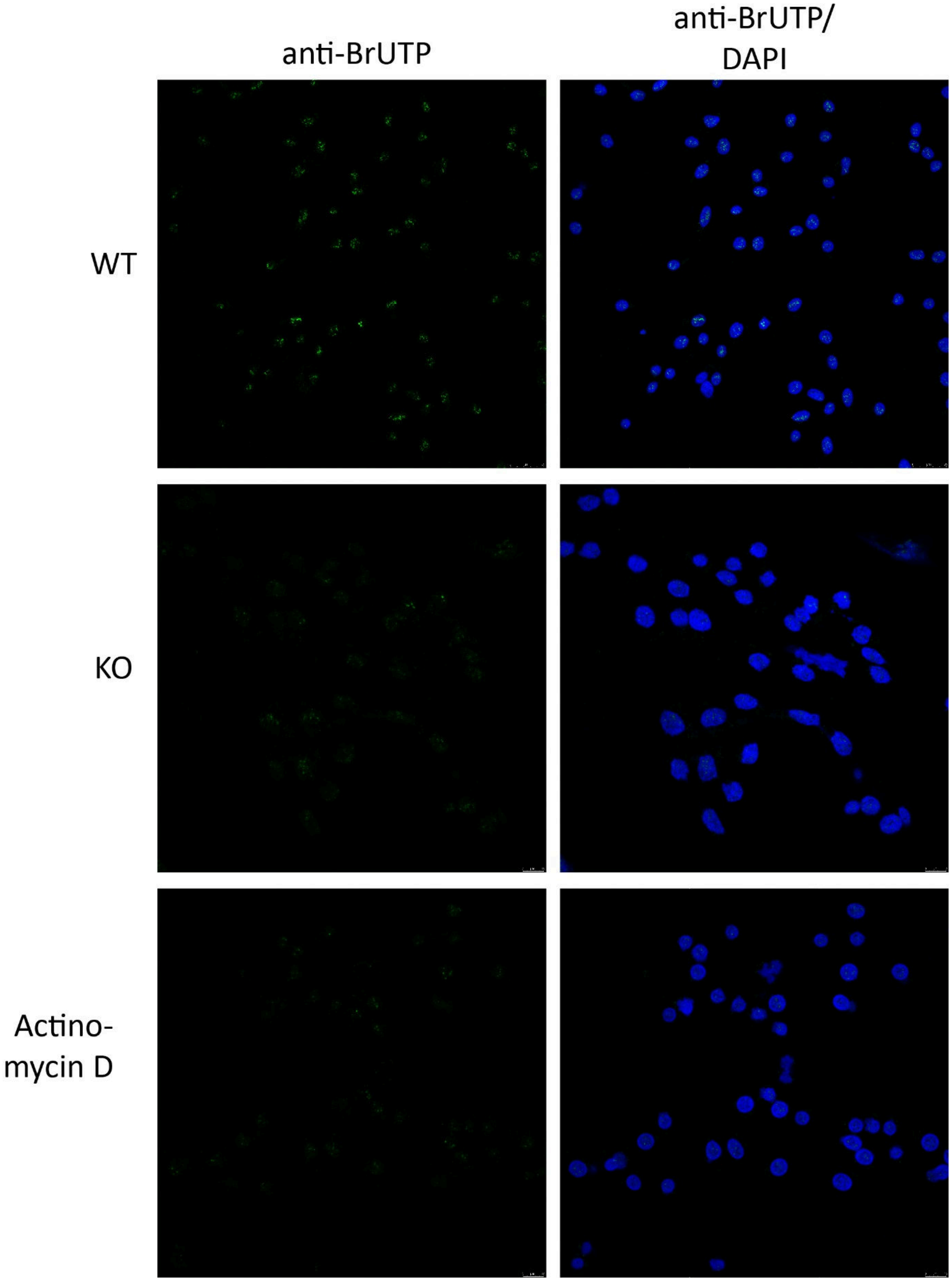

Raw data for Figure 5A

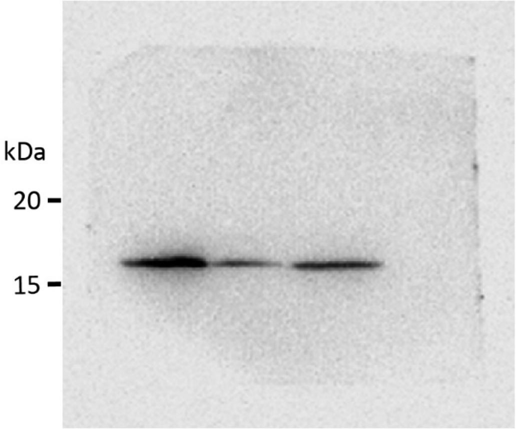

Anti-H3K9ac blot

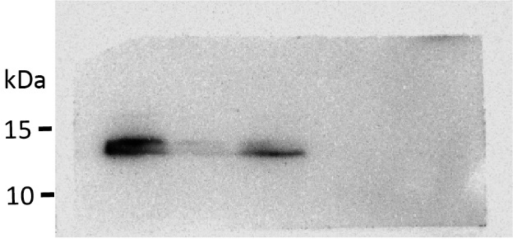

Anti-H4ac blot

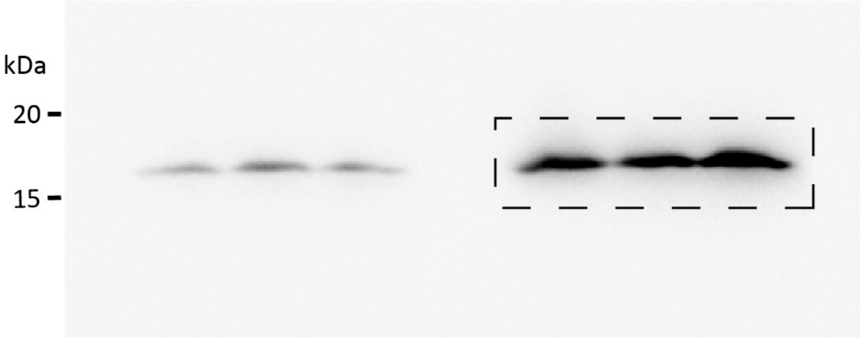

Anti-H3K4me3 blot

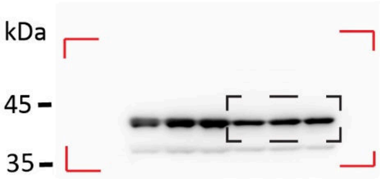

Fig. 5A. Beta-actin

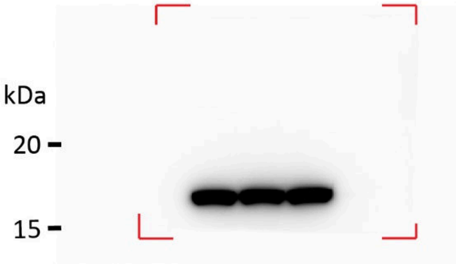

Anti-H3 blot

Note: Blots were from the same amount of samples and conducted in the same conditions of western blot. In the anti-H3K4me3 blot, the figure was cropped along the dashed rectangles.

Raw data for Figure 5E

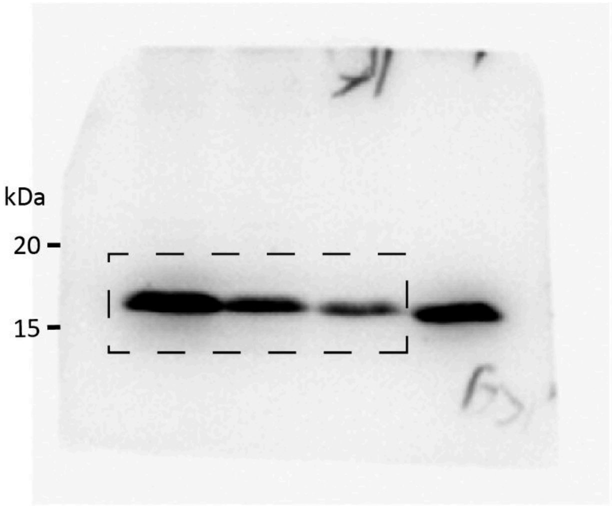

Anti-H3K9ac blot

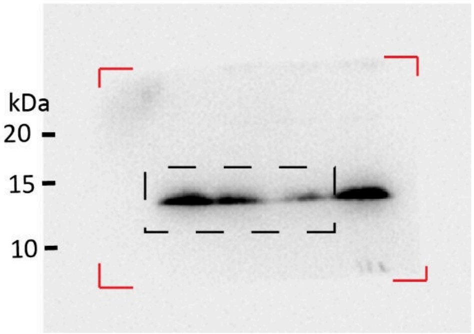

Anti-H4ac blot

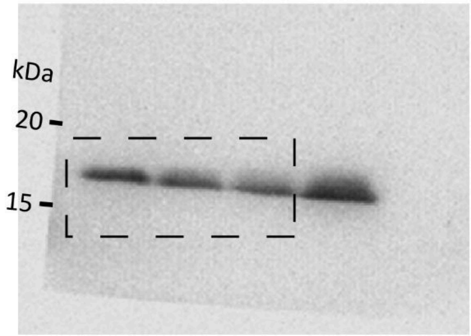

Anti-H3K4me3 blot

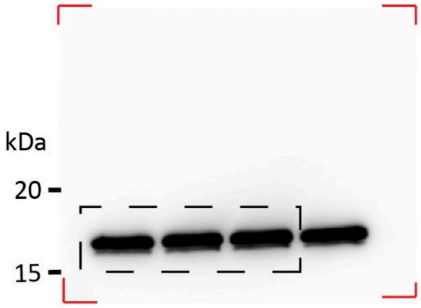

Anti-H3 blot

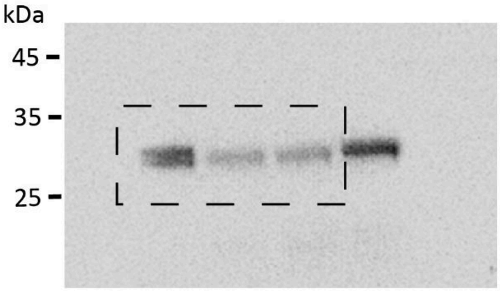

Anti-ING4 blot

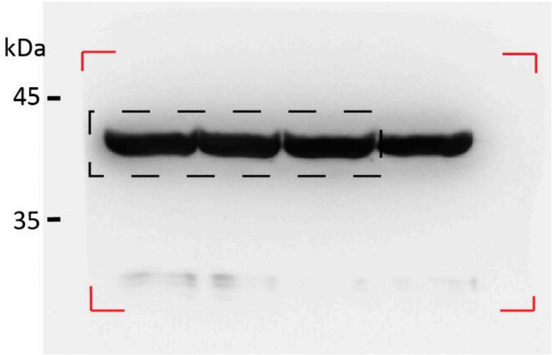

Anti-β-actin blot

Note: Blots were from the same amount of samples and conducted in the same conditions of western blot. The figures were cropped along the dashed rectangles. The red corners indicate boundaries of the blots.

Raw blots for Figure S1

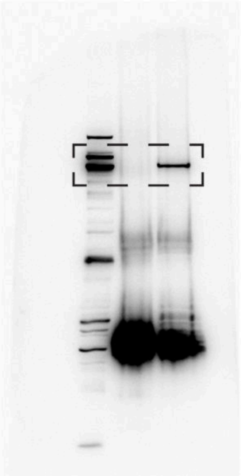

Fig. S1A. GNL2

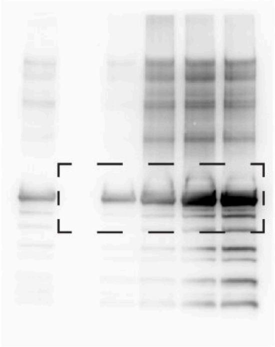

Fig. S1B. GST-GNL3

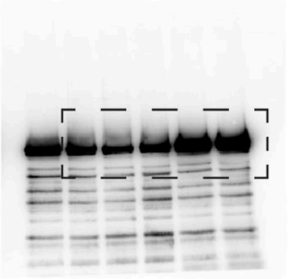

Fig. S1B. GST-NOLC1

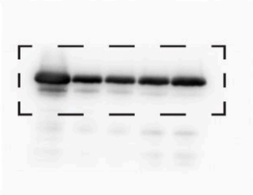

Fig. S1B. His-ING4

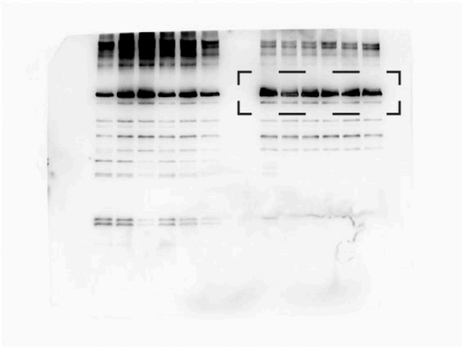

Fig. S1C. GST-GNL3

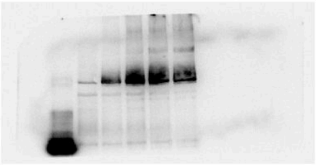

Fig. S1C. GST-NOLC1 (longer expose)

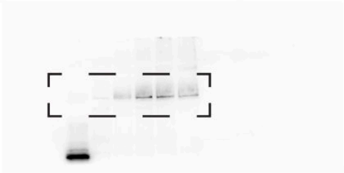

Fig. S1C. GST-NOLC1 (shorter expose)

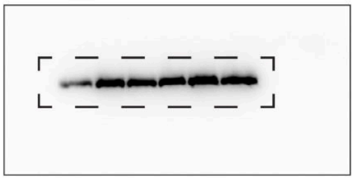

Fig. S1C. His-ING4

Note: The figures were cropped along the dashed rectangle.

Raw blots for Figure S2

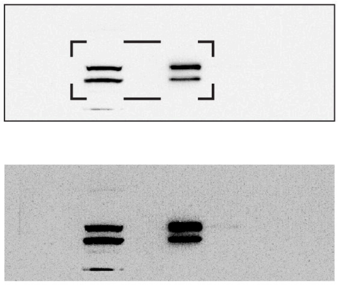

Fig. S2. HBO1
(upper: shorter exposure
lower: longer exposure)

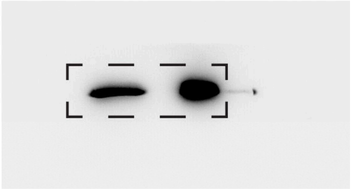

Fig. S2. Histone H3

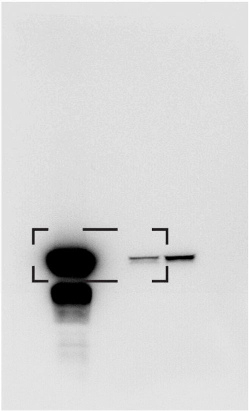

Fig. S2. p53

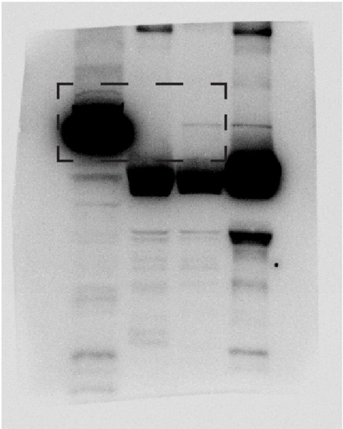

Fig. S2. p65

Note: The figures were cropped along the dashed rectangles.

Raw blots for Figure S4

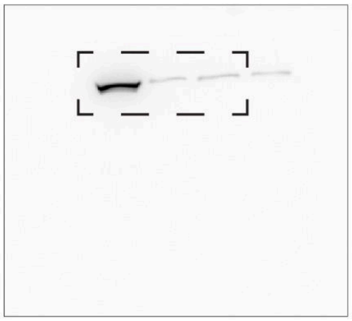

Fig. S4A. ING4

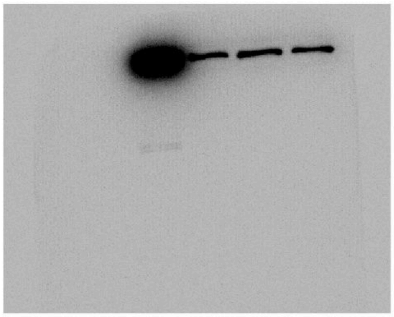

Fig. S4A. ING4
(longer expose)

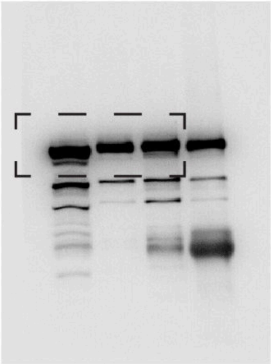

Fig. S4A. GAPDH
(reprobed membrane)

Note: Blots were from the same amount of samples and conducted in the same conditions of western blot. The figures were cropped along the rectangle.

Raw data for Figure S4B

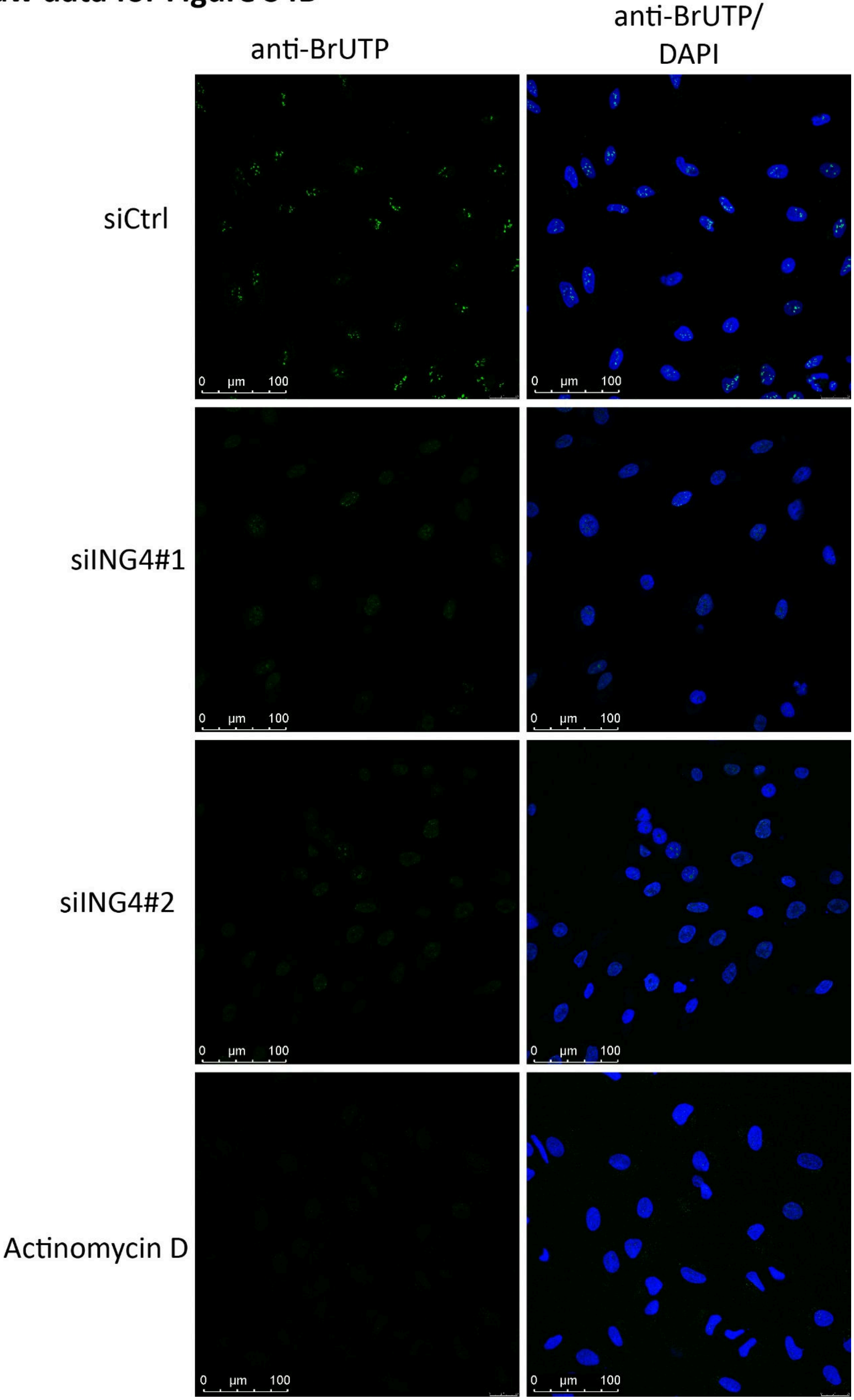

Raw blots for Figure S5

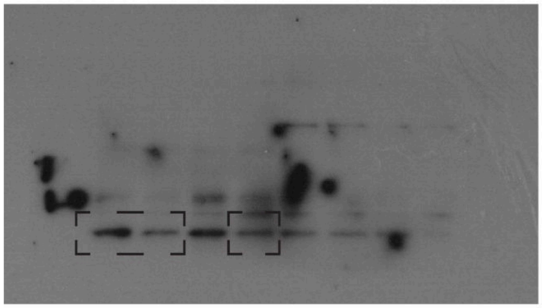

Fig. S5. ING4 (shorter expose)

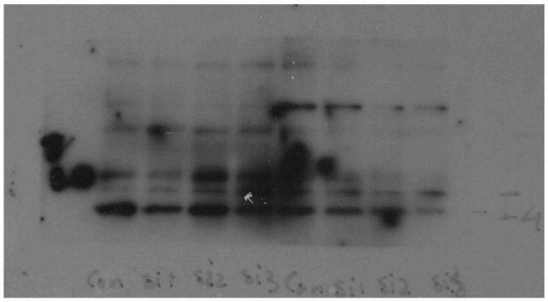

Fig. S5. ING4 (longer expose)

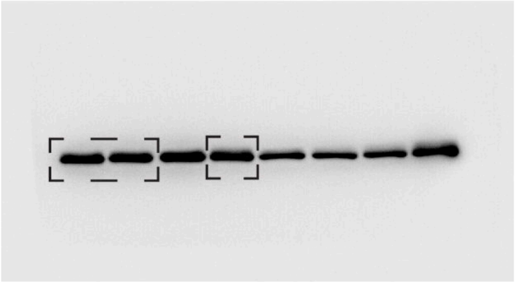

Fig. S5. GAPDH

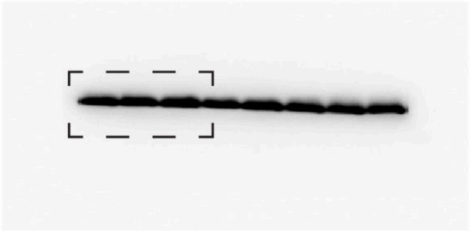

Fig. S5. Histone H3

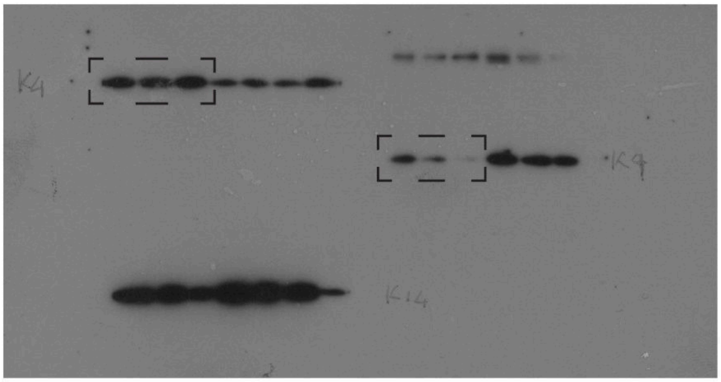

Fig. S5. H3K4me3 and H3K9ac

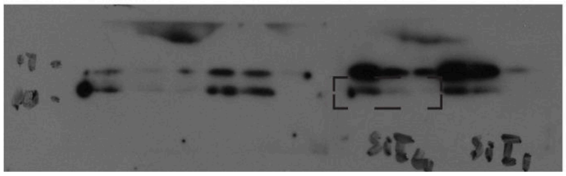

Fig. S5. H4ac

Note: Blots were from the same amount of samples and conducted in the same conditions of western blot. The figures were cropped along the dashed rectangle.
